# An Effective Treatment of Perimenopausal Syndrome by Combining Two Traditional Prescriptions of Chinese Botanical Drugs

**DOI:** 10.3389/fphar.2021.744409

**Published:** 2021-10-25

**Authors:** Junjie Lan, Caiming Wu, Wen’na Liang, Jianying Shen, Zewei Zhuo, Liu Hu, Luwei Ruan, Pengheng Zhang, Xiangrong Ye, Leqin Xu, Chengfu Li, Shengyuan Lin, Chuanhui Yang, Siqi Wu, Yingjun Dong, Haixia Ren, Huiying Huang, Bizhen Gao, Hongwei Yao, Tianwei Lin, Xueqin Chen, Candong Li

**Affiliations:** ^1^ State Key Laboratory of Cellular Stress Biology, Innovation Center for Cell Signaling Network, State-Province Joint Engineering Laboratory of Targeted Drugs From Natural Products, School of Life Sciences, Xiamen University, Cancer Research Center of Xiamen University, Xiamen, China; ^2^ Research Base of TCM syndrome, Fujian University of Traditional Chinese Medicine, Fuzhou, China; ^3^ Xiamen Hospital of Traditional Chinese Medicine Affiliated to Fujian University of Traditional Chinese Medicine, Xiamen, China; ^4^ First Hospital Affiliated to Fujian University of Medicine, Fuzhou, China; ^5^ Institute of Molecular Enzymology, School of Biology and Basic Medical Sciences, Soochow University, Suzhou, China; ^6^ The First Affiliated Hospital of Xiamen University, Xiamen, China

**Keywords:** perimenopausal syndrome, Chinese traditional medicine, phytotherapy, metabolomics, saturation transfer difference, ethnopharmacology

## Abstract

**Ethnopharmacological relevance:** Two types of traditional Chinese formulas of botanical drugs are prescribed for treating perimenopausal syndrome (PMS), a disorder in middle-aged women during their transition to menopause. One is for treating PMS as kidney deficiency (KD) due to senescence and declining reproductive functions, and the other is for treating it as liver qi stagnation (LQS) in association with stress and anxiety. Despite the time-tested prescriptions, an objective attestation to the effectiveness of the traditional Chinese treatment of PMS is still to be established and the associated molecular mechanism is still to be investigated.

**Materials and methods:** A model for PMS was generated from perimenopausal rats with chronic restraint stress (CRS). The effectiveness of traditional Chinese formulas of botanical drugs and a combination of two of the formulas was evaluated based on ^1^H NMR plasma metabolomic, as well as behavioral and physiological, indicators. To investigate whether the formulas contained ligands that could compensate for the declining level of estrogen, the primary cause of PMS, the ligand-based NMR technique of saturation transfer difference (STD) was employed to detect possible interacting molecules to estrogen receptors in the decoction.

**Results:** Each prescription of the classical Chinese formula moderately attenuated the metabolomic state of the disease model. The best treatment strategy however was to combine two traditional Chinese formulas, each for a different etiology, to adjust the metabolomic state of the disease model to that of rats at a much younger age. In addition, this attenuation of the metabolomics of the disease model was by neither upregulating the estrogen level nor supplementing an estrogenic compound.

**Conclusion:** Treatment of PMS with a traditional Chinese formula of botanical drugs targeting one of the two causes separately could ameliorate the disorder moderately. However, the best outcome was to treat the two causes simultaneously with a decoction that combined ingredients from two traditional prescriptions. The data also implicated a new paradigm for phytotherapy of PMS as the prescribed decoctions contained no interacting compound to modulate the activity of estrogen receptors, in contrast to the treatment strategy of hormone replacement therapy.

## Introduction

Perimenopause is a transition state in middle-aged women with gradually declining ovarian function and irregular menstrual cycles (Zhang et al., 2013; Gemmell et al., 2017; Marques-Lopes et al., 2017; Zeng et al., 2018). With erratically fluctuating and descending estrogen level in the body, approximately 40–60% of perimenopausal women suffer from perimenopausal syndrome (PMS) characterized by disorders in the endocrine system and autonomic nervous system (Li et al., 2013; Xu et al., 2019). Clinical symptoms of PMS are diverse and include hot flashes, night sweats, insomnia, depression, irritability, fatigue, and cognitive impairment (Stearns et al., 2002; Elkins et al., 2008). The incidence of cardiovascular diseases, such as hypertension, atherosclerosis, myocardial infarction, and cerebral hemorrhage, increases in perimenopausal women with PMS (Nelson, 2008). The incidence of depression and osteoporosis is also higher in perimenopausal women with PMS than in healthy women (Whiteley et al., 2013).

The diagnosis of traditional Chinese medicine (TCM) is based on symptom-based pattern differentiation (also known as symptom differentiation, Zheng differentiation, pattern diagnosis, and pattern classification) (Liang et al., 2009; Liang et al., 2011; Li et al., 2015). Over the centuries of TCM practice, PMS is diagnosed as kidney deficiency (KD), a jargonistic term for the debility of bodily functions and loss of vitality, from senescence. Symptoms of insomnia, night sweat, lack of libido, back pain, and declining vitality, among others, were signs compatible with those of KD (Liang et al., 2009; Chen et al., 2020). However, KD does not seem to be the sole cause of PMS. As senescence is irreversible, aging could only deepen the degree of KD for middle-aged women. It is contradictory then that the symptoms of PMS will be naturally relieved in the later stage of perimenopause and after menopause as women get older. It is an indication that KD might not be the only cause for PMS. Obviously, other factors need to be taken into account in the diagnosis of PMS for better treatment.

The symptoms of hot flash, night sweat, insomnia, depression, irritability, fatigue, and cognitive impairment, which occur frequently in women suffering PMS, are compatible with those of liver qi stagnation (LQS), a diagnostic designation for illness correlated with anxiety and stress in pattern differentiation (Liang et al., 2010; Yu et al., 2018). There were demonstrations that prescriptions of decoctions for LQS were efficacious for treating PMS (Liang et al., 2010; Chen et al., 2014; Li et al., 2014; Li et al., 2015). For better prescription to treat PMS, it is important to investigate the physiological parameters for treating PMS as either KD, LQS, or a combination of both.

Herein, a rat model for PMS was generated with aging and stress. ^1^H NMR metabolomics of plasma samples showed that a combined decoction for treating both KD and LQS could restore the metabolomic condition of the PMS rat model to that of rats at a younger age before entering the perimenopausal period. The data also indicated that the treatment adopted here modulated the metabolic state of the PMS rat without either altering the serum level of estrogen or supplementing a constituent capable of interacting with an estrogen receptor to attenuate its activity, implicating that TCM treatment of PMS might be under a different paradigm from the typical treatment strategy of hormone replacement therapy which relieves the symptoms of PMS by supplementing an estrogenic compound to modulate the activity of estrogen receptors.

## Methods

### Preparing the Decoctions

The botanical drugs used to prepare the decoctions were purchased from the Third People’s Hospital of Fujian University of Traditional Chinese Medicine (FJUTCM). The botanical drugs were authenticated by the staff in the Herb Identification, Teaching and Research Division of the College of Pharmacy of FJUTCM. The voucher specimens were kept in the College of Pharmacy of FJUTCM, Fujian, China. The decoctions, including those of Chaihu-Shugan-San (CSS), Yougui, and Zuogui, were prepared according to the Pharmacopoeia of the People’s Republic of China (2015 Edition). The combined decoction of Zuogui and CSS (Zuogui-CSS) was also prepared. The ingredients are listed in Tables 1–4.

**TABLE 1:** The herbal composition of Chaihu-Shugan-San (CSS).

Chinese name	Botanical name (family name)	Pharmaceutical name	Amount (g)	Voucher specimens
Chai Hu	*Bupleurum chinense* DC. (Apiaceae)	Bupleuri radix	6	1000000618
Bai Shao	*Paeonia lactiflora* Pall. (Paeoniaceae)	Paeoniae radix alba	4.5	1000000608
Zhi Ke	*Citrus aurantium* L. (Rutaceae)	Aurantii fructus	4.5	1000000742
Chuan Xiong	*Ligusticum striatum* DC. (Apiaceae)	Chuanxiong rhizoma	4.5	1000000872
Chen Pi	*Citrus reticulata* Blanco (Rutaceae)	Citri reticulatae pericarpium	6	1000000674
Xiang Fu	*Cyperus rotundus* L. (Cyperaceae)	Cyperi rhizoma	4.5	1000001080
Zhi Gan Cao	*Glycyrrhiza uralensis* Fisch. (Fabaceae)	Glycyrrhizae radix et rhizoma praeparata cum melle	1.5	1000000884

**TABLE 2:** The herbal composition of Yougui.

Chinese name	Botanical name (family name)	Pharmaceutical name	Amount (g)	Voucher specimens
Shu Di Huang	*Rehmannia glutinosa* (Gaertn.) DC. (Plantaginaceae)	Rehmanniae radix praeparata	9	1000001065
Shan Yao	*Dioscorea oppositifolia* L*.* (Dioscoreaceae)	*Dioscorea* rhizome	9	1000000908
Shan Zhu Yu	*Cornus officinalis* Siebold & Zucc. (Cornaceae)	Corni fructus	6	1000001097
Gou Qi Zi	*Lycium barbarum* L*.* (Solanaceae)	Lycii fructus	9	1000001087
Zhi Gan Cao	*Glycyrrhiza uralensis* Fisch. (Fabaceae)	Glycyrrhizae radix et rhizome	3	1000000884
Du Zhong	*Eucommia ulmoides* Oliv*.* (Eucommiaceae)	Eucommiae cortex	9	1000000792
Rou Gui	*Cinnamomum cassia* (L.) J.Presl (Lauraceae)	Cinnamomi cortex	3	1000001007
Fu Zi	*Aconitum carmichaeli* Debeaux (Ranunculaceae)	Aconiti lateralis radix praeparata	6	1000000606

**TABLE 3:** The herbal composition of Zuogui.

Chinese name	Botanical name (family name)	Pharmaceutical name	Amount (g)	Voucher specimens
Shu Di Huang	*Rehmannia glutinosa* (Gaertn.) DC. (Plantaginaceae)	Rehmanniae radix praeparata	9	1000001065
Shan Yao	*Dioscorea oppositifolia* L*.* (Dioscoreaceae)	*Dioscorea* rhizome	6	1000000908
Shan Zhu Yu	*Cornus officinalis* Siebold & Zucc. (*Cornace*ae)	Corni fructus	6	1000001097
Gou Qi Zi	*Lycium barbarum L.* (Solanaceae)	Lycii fructus	6	1000001087
Zhi Gan Cao	*Glycyrrhiza uralensis* Fisch. (Fabaceae)	Glycyrrhizae radix et rhizome praeparata cum melle	3	1000000884
Fu Ling	*Poria cocos* ^a^ (Polyporaceae)	Poria	4.5	1000001020

a *Poria cocos* is the dried sclerotia of *Wolfiporia cocos* (F. A. Wolf) Ryvarden & Gilb.

**TABLE 4:** The herbal composition of Zuogui-CSS.

Chinese name	Botanical name (family name)	Pharmaceutical name	Amount (g)	Voucher specimens
Chai Hu	*Bupleurum chinense* DC. (Apiaceae)	Bupleuri radix	6	1000000618
Bai Shao	*Paeonia lactiflora* Pall. (Paeoniaceae)	Paeoniae radix alba	4.5	1000000608
Zhi Ke	*Citrus* × *aurantium* L. (Rutaceae)	Aurantii fructus	4.5	1000000742
Chuan Xiong	*Ligusticum striatum* DC (Apiaceae)	Chuanxiong rhizoma	4.5	1000000872
Chen Pi	*Citrus reticulata* Blanco (Rutaceae)	Citri reticulatae pericarpium	6	1000000674
Xiang Fu	*Cyperus rotundus* L. (Cyperaceae)	Cyperi rhizoma	4.5	1000001080
Zhi Gan Cao	*Glycyrrhiza uralensis* Fisch. (Fabaceae)	Glycyrrhizae radix et Rhizoma praeparata cum melle	4.5	1000000884
Shu Di Huang	*Rehmannia glutinosa* (Gaertn.) DC. (Plantaginaceae)	Rehmanniae radix praeparata	9	1000001065
Shan Yao	*Dioscorea oppositifolia* L*.* (Dioscoreaceae)	*Dioscorea* rhizoma	6	1000000908
Shan Zhu Yu	*Cornus officinalis* Siebold & Zucc. (Cornaceae)	Corni fructus	6	1000001097
Gou Qi Zi	*Lycium barbarum* L*.* (Solanaceae)	Lycii fructus	6	1000001087
Fu Ling	*Poria cocos* ^a^ (Polyporaceae)	Poria	4.5	1000001020

a *Poria cocos* is the dried sclerotia of *Wolfiporia cocos* (F. A. Wolf) Ryvarden & Gilb.

For preparing the decoctions, botanical drugs for each formula were mixed and macerated in distilled water at room temperature for 1 h. The mixture was then decocted twice with distilled water for half of an hour, at a ratio of 1:10 w/v and at a ratio of 1:5 w/v, respectively. The two resultant decoctions were combined, centrifuged, and filtrated. The decoctions of CSS, Yougui, Zuogui, and Zuogui-CSS were concentrated to 0.2777, 0.476, 0.304, and 0.5817 g/ml of the crude drugs, respectively. All decoctions were stored at −80°C.

### Generation of the Rat Model for PMS With KD and LQS

SPF-grade (specific pathogen-free grade) female Sprague-Dawley (SD) rats (5 months old) were purchased from Shanghai Xipuer-Bikai Laboratory Animal Co., Ltd. (Shanghai, China). Animals were housed in individually ventilated cages and kept in an environmentally controlled room with a 12 h light/dark cycle at constant temperature (23°C) and humidity (55%). The rats had free access to the standard laboratory water and diet.

Perimenopausal rats were generated based on previous studies (Cai et al., 2016; Chen et al., 2020). These rats were with irregular menstrual cycles at the age of about 11–13 months. Forty of these perimenopausal rats were selected and randomly divided into five groups with eight rats each. The rat model with KD and LQS was generated by the method of chronic restraint stress (CRS) (Liang et al., 2015). The food intake, water intake, body weight, appearance, and activity of the rats were monitored weekly throughout the experiment. Plasma concentrations of estrogen (E2), adrenocorticotropic hormone (ACTH), cortisol (CORT), corticotropin-releasing hormone (CRH), 5-hydroxytryptamine (5-HT), dopamine (DA), and beta-endorphin (β-EP) were determined by ELISA (with kits from CUSABIO, China).

The human equivalent dose (HED) for rat has a multiplication factor of 6:37 (Reagan-Shaw et al., 2008). So the final concentration of crude drugs for Yougui, Zuogui, CSS, and Zuogui-CSS was adjusted to 0.476, 0.304, 0.2777, and 0.5817 g/ml, respectively. The decoctions were administered by gavage with a feeding tube once daily for 4 weeks at a dose of 1 ml for every 100 g of body weight. After overnight fasting, blood samples were collected and centrifuged. All plasma samples were stored at −80°C.

### Sample Preparations and ^1^H NMR Data Acquisition

The NMR spectra were acquired at 298 K on a 600 MHz Bruker AVANCE II NMR spectrometer (Bruker BioSpin, Rheinstetten, Germany) operating at 600.13 MHz for ^1^H signals. The ^1^H NMR spectrum was acquired using a Carr-Purcell-Meiboom-Gill (CPMG, RD-90-(τcp-180-τcp)-acquisition) spin-echo pulse sequence to suppress the water with a total spin-spin relaxation delay (RD) of 320 ms to attenuate broad signals from proteins and lipoproteins due to their short transverse relaxation time. Each ^1^H NMR spectrum was obtained with 80 scans with a spectral width of 12335.5 Hz, spectral size of 65,536 points, pulse width (PW) of 30° (12.7 μs), and RD of 2.0 s. The FIDs were Fourier transformed with LB = 0.3 Hz.

The ^1^H NMR spectra were manually corrected for phase and baseline with the Topspin 3.2 software. Integrations of water resonance (4.70–5.15 ppm) in the spectra of aqueous samples were excluded. The metabolites were normalized to the total integrated spectral area (−0.55–8.55 ppm) for aqueous samples. The data sets were log-transformed and Pareto-scaled (mean-centered and divided by the square root of the standard deviation of each variable) prior to statistical analysis.

### Multivariate Analysis

Multivariate analyses of the NMR spectra were carried out with the algorithm of PLS-DA (Partial Least Square Discriminant Analysis) implemented in MetaboAnalyst 4.0 (Chong et al., 2018). The model quality was validated based on two parameters, R^2^ (goodness-of-fit parameter) and Q^2^ (predictive ability). A model with a large *R* ^2^ (close to 1) and Q^2^ (Q^2^ ≥ 0.5) was considered an excellent model. The PLS-DA model was also validated by the permutation test in which the class membership was randomly shuffled by 100 times for calculating the response values. The new *R* ^2^ and Q^2^ values were lower than the original ones indicating that the model was not overfitting (Chang et al., 2007).

The important metabolites were identified based on their respective variable influence on projection (VIP) score in the PLS-DA analysis. Significant differences of the selected signals of the main metabolites, which were responsible for class discrimination, were analyzed using a *t*-test in GraphPad Prism 5 software. The data are presented as *p <* 0.05 (*), *p <* 0.01 (**), and *p <* 0.001 (***).

### Protein Production and Saturation Transfer Difference (STD) NMR Experiment

The human ligand-binding domain of estrogen receptor α (ERα LBD) containing residues 297–554 in the ERα sequence was expressed in *E.coli* with BL21 (DE) cells and a pET-22b vector. It was purified following an established protocol (Bruning et al., 2010). STD NMR (Mayer and Meyer, 2001; Meyer and Peters, 2003) was employed to detect the ERα LBD interaction with small molecules in the TCM decoctions at 298 K on an 850 MHz Bruker ADVANCE III spectrometer (Bruker BioSpin, Rheinstetten, Germany) equipped with a TCI CryoProbe. The RD was set to 2 s. Selective on-resonance irradiation frequency was set to 0.47 ppm with a saturation time of 2 s. The selective saturation was achieved by a train of 50 ms Gauss-shaped pulses separated by a 2 ms delay. The duration of the presaturation of 2 s was adjusted using *n* = 128 cycles. Off-resonance irradiation frequency for the reference spectrum was applied at 50 ppm. The decoction of Zuogui-CSS was mixed with the recombinant ERα LBD (20 μM) in phosphate buffer (50 mM sodium phosphate, pH 7.4, 150 mM NaCl, and 5% glycerol) in the presence of 10% D_2_O (Cambridge Isotope Laboratories, United States). The NMR data were analyzed with the Topspin 3.2 software.

### *In Vitro* Gastrointestinal Digestion

Simulated gastric fluid (SGF) and simulated intestinal fluid (SIF) were prepared following the protocols in Pharmacopoeia of the People’s Republic of China (2015 Edition). SGF was prepared by mixing 16.40 ml of diluted hydrochloric acid, 800 ml of Milli-Q water, and 10 g of pepsin (Sigma-Aldrich, P7012) to a final volume of 1,000 ml. SGF thus prepared had a pH value of about 1.4. Solution A for SIF was prepared by dissolving 6.80 g of potassium dihydrogen phosphate in Milli-Q water to a final volume of 500 ml. The pH value of solution A was adjusted to 6.8 with a NaOH solution (0.1 mol/L). Solution B for SIF was prepared by dissolving 10 g of pancreatin (Sigma-Aldrich, P7545) in Milli-Q water. Solutions A and B were mixed and the final volume was adjusted to 1,000 ml with Milli-Q water to make SIF.

The SGF-treated Zuogui-CSS was prepared by incubating Zuogui-CSS with SGF at a ratio of 1:10 at 37°C with shaking at 100 rpm for 2 h. The sample was then dried using a rotary evaporator at 70°C. The SGF-treated samples were rehydrated with Milli-Q water. Similarly, Zuogui-CSS was treated with SIF by mixing Zuogui-CSS and SIF in a ratio of 1:4 for a 4 h incubation. It was similarly dried and then rehydrated as for the SGF-treated samples. Zuogui-CSS was also sequentially treated with SGF for 2 h and SIF for 4 h. The SGF- and SIF-treated samples were analyzed by ^1^H NMR spectroscopy.

## Results

### Perimenopause Was Accompanied by Metabolomic Changes

The perimenopausal rats (PM rats) were with irregular menstrual cycles starting at the age of about 12 months old and these rats were deemed to be with KD (Miao et al., 2018; Chen et al., 2020). The weight, sugar preference rate, and behavior indices of rats were of no significant difference between 6-month-old rats and perimenopausal rats, based on sugar preference test (SPT) and open-field test (OFT) (Strekalova et al., 2004; Kraeuter et al., 2019) (Supplementary Figure S1). While the levels of CRH, CORT, β-EP, and 5-HT showed no significant difference, the levels of E2 and DA were lower in perimenopausal rats, whereas the level of ACTH was upregulated (Supplementary Figure S2). These data were compatible with the physiological state of the perimenopausal rats (Cai et al., 2007; Fu et al., 2017).

In the preceding study, the metabolic profiles of rats entering the perimenopausal state and that of younger age were clearly different with metabolites for energy metabolism, such as lipid, glucose, trimethylamine-n-oxide, glutamine, pyruvate, acetoacetate, citrate, betaine, and acetone, influencing the shift of metabolomes (Chen et al., 2020).

### Influence of LQS on Perimenopausal Rats

**FIGURE 1:**
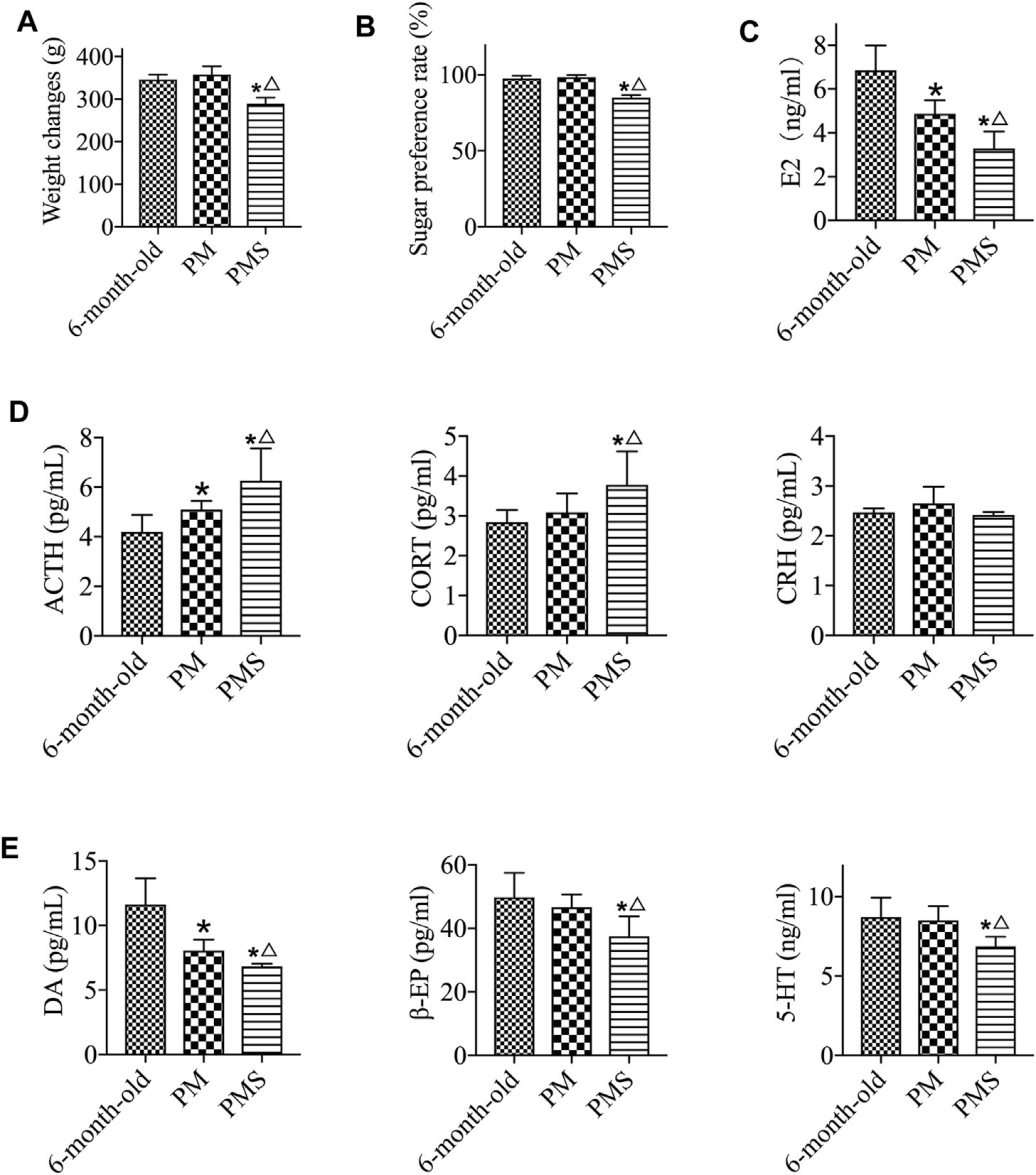
PMS rats. **(A)** The body weights of 6-month-old, PM, and PMS rats. The PMS rats were with significant weight loss. **(B)** The PMS rats were of decreased sugar preference rate. **(C)** The level of estrogen in PMS rats was further decreased from that of perimenopausal rats. **(D)** The levels of ACTH and CORT in PMS rats were enhanced, while the level of CRH was not significantly altered. **(E)** The levels of β-EP, 5-HT, and DA were all lowered in PMS rats. “*” indicates the comparison with the 6-month-old rats [*p* < 0.05 (*)]; “△” indicates the comparison with the perimenopausal rats [*p* < 0.05 (△)].

To generate a rat model for PMS, the perimenopausal rats were treated with CRS. These rats were considered to be with both KD and LQS (Li et al., 2015; Miao et al., 2018). The PMS rats were with a weight loss (Figure 1A), decreased sugar preference rate (Figure 1B), and reduced level of activity compared to either the 6-month-old or the PM rats without the stress (Supplementary Figure S3). The analyses also showed that the concentrations of E2, DA, β-EP, and 5-HT in the peripheral blood of the PMS rats decreased rapidly (Figures 1C–E), while the levels of ACTH and CORT in direct correlation with the HPA axis reactivity were elevated. These data indicated that PMS rats were under stress and depression.

**FIGURE 2:**
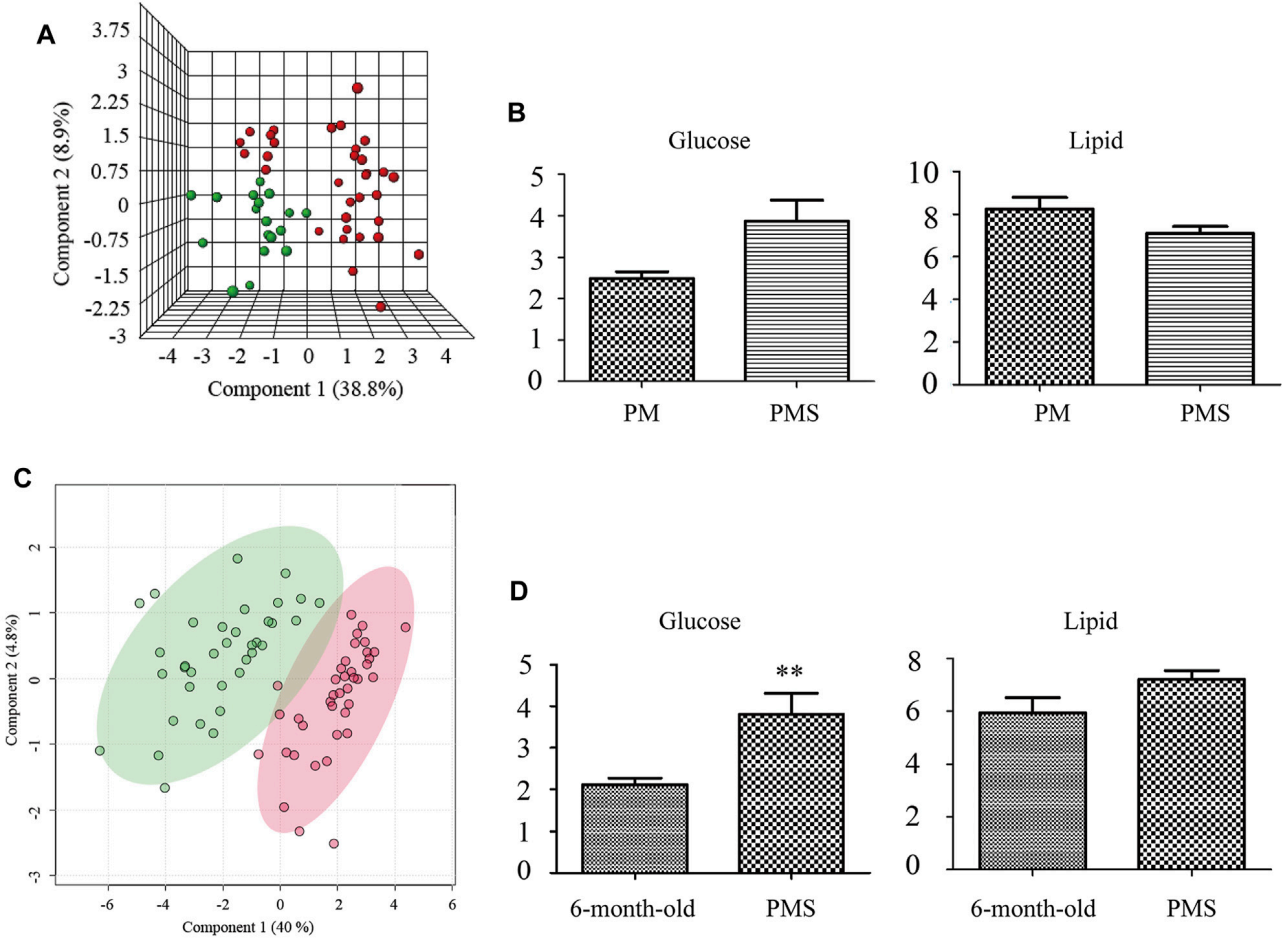
Metabolomic profiles of 6-month-old, PM and PMS rats. **(A)** 3D representation of PLS-DA analysis of the PM (green) and PMS rats (red). The metabolomic profiles of the PMS rats changed significantly. **(B)** Transition from perimenopause to PMS, the levels of glucose were upregulated, while the levels of lipid were downregulated. **(C)** PLS-DA analysis of metabolomic profile from the plasma of 6-month-old rats (green) and PMS rats (red). **(D)** Compared with 6-month-old rats, the levels of glucose and lipid were upregulated. *p* < 0.01 (**).

The metabolomic states between PM rats and PMS rats were distinctly different (Figure 2A) and the metabolites that dictated the metabolomic change in the PMS rats were glucose and lipid (Supplementary Figure S4 and Figure 2B). The levels of creatine, glycerol, trimethylamine-n-oxide, betaine, glutamine, pyruvate, acetoacetate, methionine, and glycoproteins increased in the plasma of PMS rats. In addition, the concentrations for unsaturated lipid, glycine, acetone, acetate, and isoleucine decreased (Supplementary Figure S5 and Supplementary Table S1).

The metabolomic profile of PMS rats was also distinct from that of the 6-month-old rats (Figure 2C) with elevated levels of glucose, creatine, trimethylamine N-oxide, betaine, choline, creatinine, citrate, glutamine, succinate, pyruvate, acetoacetate, methionine, glycoprotein, acetate, and valine, but a downregulated level of glycine (Figure 2D, Supplementary Figures S6, S7, and Supplementary Table S2).

The above data indicated that the glucose metabolism and lipid metabolism of perimenopausal rats were upregulated from those of the 6-month-old rat. Entering the LQS state, the glucose metabolism was upregulated but the lipid metabolism was suppressed in PMS rats comparing to the perimenopausal rats. This aberrant state of glucose metabolism was in line with the fact that the rats were under stress and LQS was associated with downregulated lipid metabolism.

### Influence of the Decoctions for KD on PMS Rats

**FIGURE 3:**
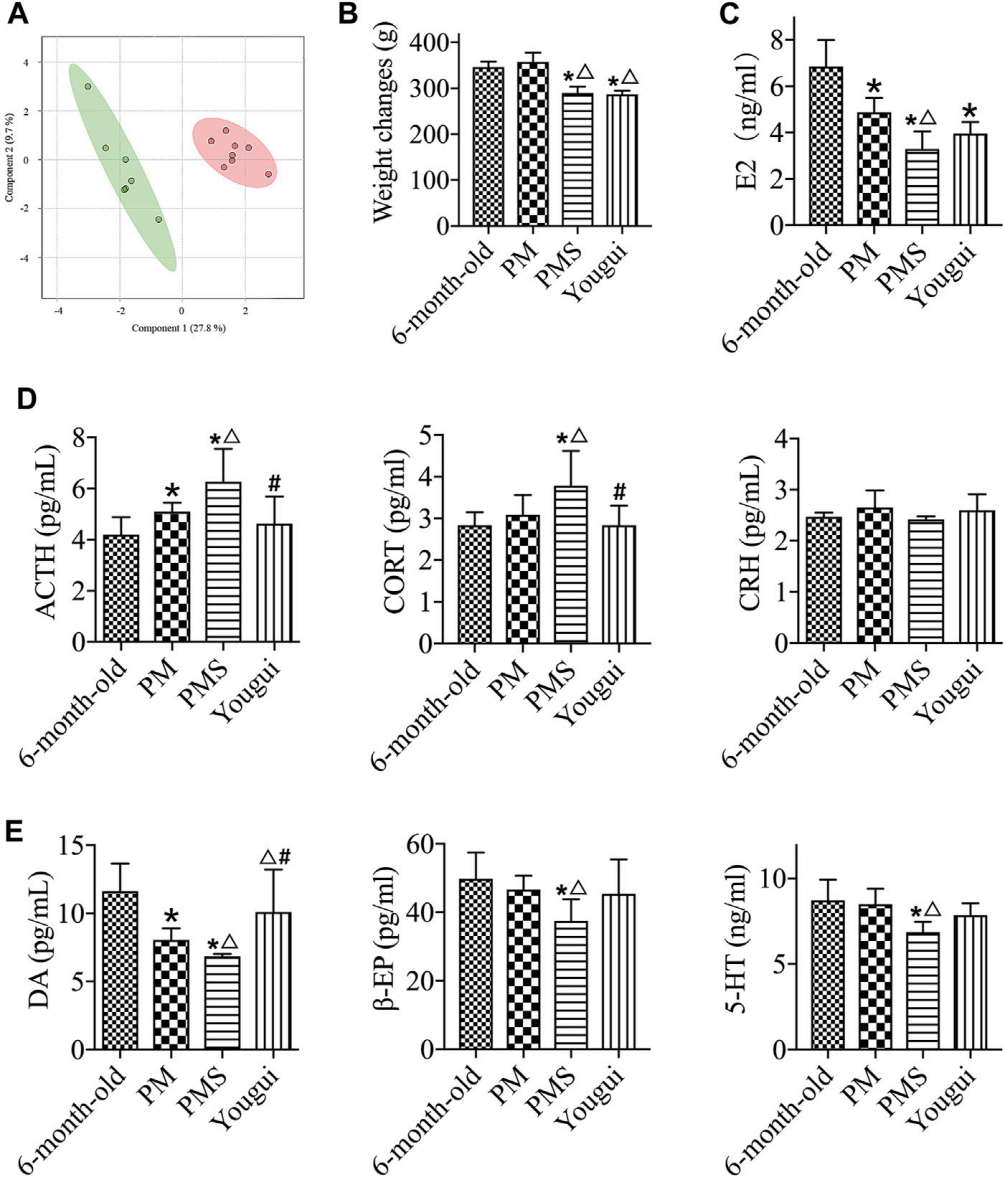
Treatment with Yougui. **(A)** The metabolomics state of PMS (red) further progressed away from the original state after 4 weeks of gavage with a saline solution (green). The metabolic state changed rapidly. **(B)** Weight changes of PMS rats treated with Yougui. There was no statistically significant difference in the weight of PMS rats before and after treatment with Yougui. **(C)** Treatment of Yougui did not result in a statistically different E2 level in PMS rats. **(D)** ELISA results of ACTH, CORT, and CRH. Compared with PMS rats, the levels of ACTH and CORT were reduced after treatment with Yougui. **(E)** The levels of DA, β-EP, and 5-HT. Compared with PMS rats, the levels of DA were enhanced after treatment with Yougui. “*” indicates the comparison with 6-month-old rats [*p* < 0.05 (*)]; “△” indicates the comparison with the perimenopausal rats [*p* < 0.05 (△)]; “#” indicates the comparison with the PMS rats [*p* < 0.05 (#)].

After entering the PMS state, the metabolomic state changed rapidly without any treatment (Figure 3A). Since PMS would be with both KD and LQS by TCM diagnosis, it would be reasonable to treat the model with the corresponding prescriptions in TCM. KD was subdivided into kidney yin deficiency, kidney yang deficiency, and the combination of the two. Traditionally, Yougui, a decoction made from a mix of Chinese botanical drugs (Table 2), is prescribed for kidney yang deficiency and Zuogui (Table 3) is prescribed for kidney yin deficiency (Zhao et al., 2011; He et al., 2014; Fu et al., 2017; Chen et al., 2019). Previously, we discovered that both decoctions of Zuogui and Yougui could adjust the metabolomic states of PM rats (Chen et al., 2020). Metabolomic regulations of PMS rats by these two decoctions were also investigated.

PMS rats were treated with the decoction of Yougui (Yougui) for comparison with rats of 6-months old and of perimenopause. The treatment of Yougui showed little influence on the weight (Figure 3B) and the level of E2 of PMS rats (Figure 3C). However, the levels of ACTH and CORT were reduced, while the level of DA increased (Figures 3D,E). Overall, following the treatment with Yougui, the weight and the abovementioned endocrine indexes of PMS rats were not significantly changed. The E2 level was not significantly different either (Figure 3).

**FIGURE 4:**
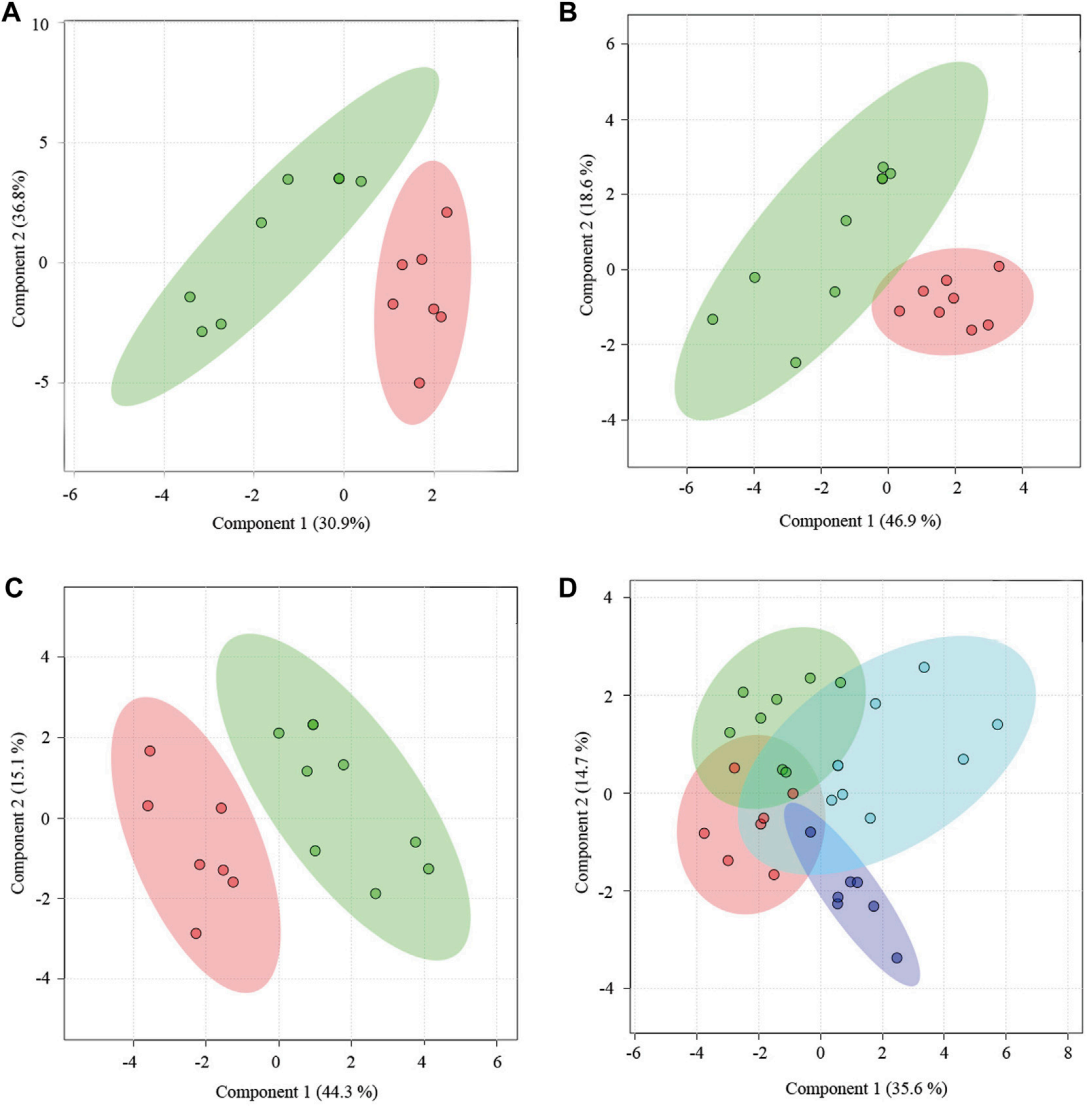
Yougui regulated the metabolomic state of PMS rats. **(A)** PLS-DA analysis of metabolomic profile from the plasma of PMS rats before (red) and after (green) treatment with Yougui. **(B)** Metabolomic profile of the plasma of Yougui-treated PMS rats (green) and perimenopausal rats (red). **(C)** Metabolomic profile from the plasma of Yougui-treated PMS rats (green) and that of 6-month-old rats (red). **(D)** PLS-DA analysis of metabolomics profiles from the plasmas of PMS rats treated with (blue) and without Yougui (purple), perimenopausal (green) rats, and 6-month-old rats (red). Yougui treatment modulated the metabolomic state of PMS rats towards that of rats at younger ages.

Yougui treatment did alter the metabolomic state of PMS rats by bringing the metabolomic state of PMS rats closer to that of perimenopausal rats (Figures 4A,B). The metabolomic difference between PMS rats treated with Yougui and rats of 6 months old though was still significant (Figure 4C). Overall, the data indicated that Yougui treatment improved the metabolomic state of PMS rats and brought its metabolomic profile closer to those of perimenopausal rats without LQS (Figure 4D). The dominant metabolites altering the metabolomic profile in the Yougui treatment were lipid, glucose, amino acids, and proteins (Supplementary Figures S8–S10).

After Yougui treatment, the levels of acetate and isoleucine increased significantly in the PMS rats, but the level of glucose decreased. In comparison to the 6-month-old rats, the levels of creatine, trimethylamine-n-oxide, choline, creatinine, glutamine, acetoacetate, glycoprotein, acetate, valine, isoleucine, and leucine were significantly higher (Supplementary Figure S11 and Supplementary Table S3).

**FIGURE 5:**
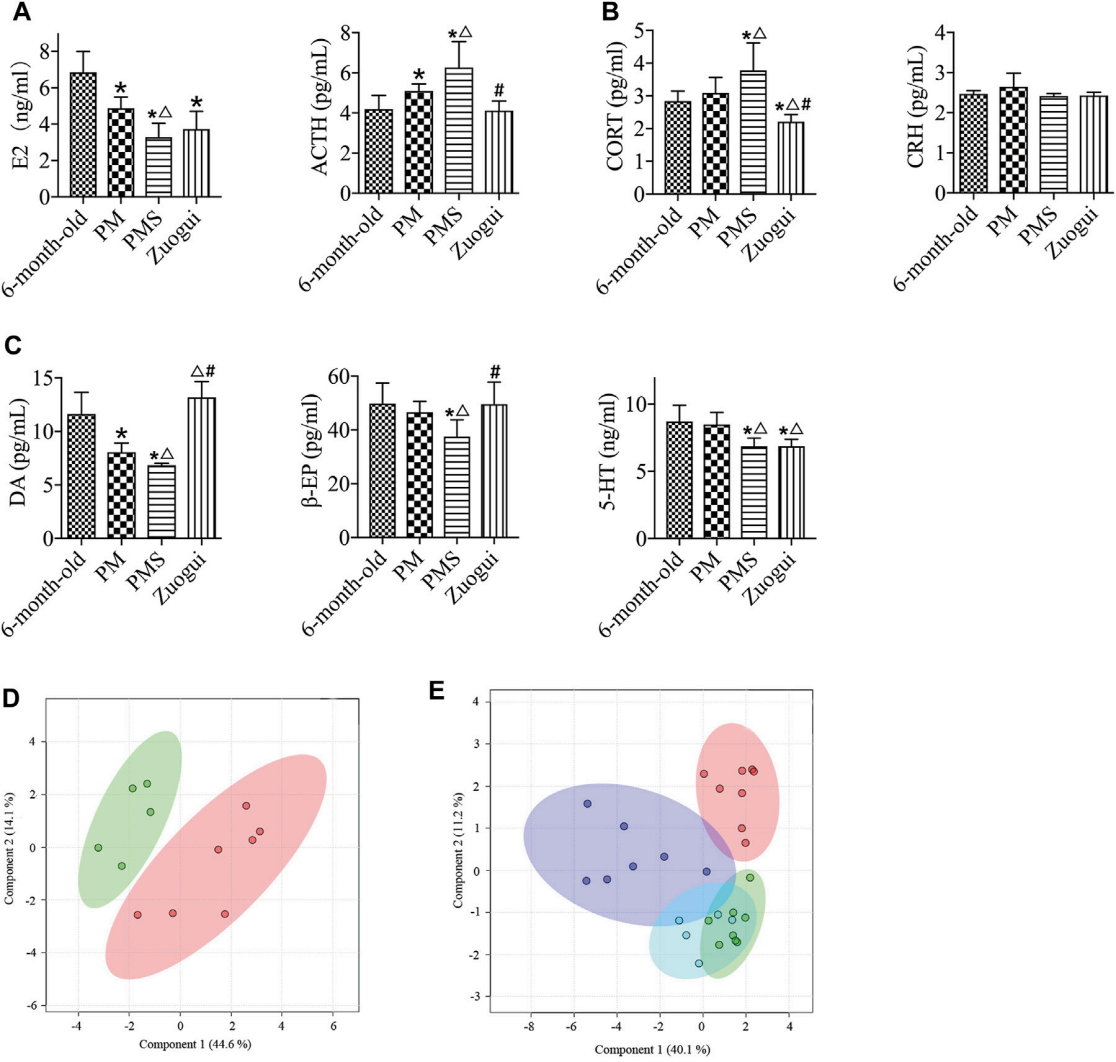
Zuogui regulated the levels of metabolites and endocrine indexes in PMS rats. **(A)** The E2 levels of PMS rats treated Zuogui were not significantly different. **(B)** The levels of ACTH and CORT of PMS rats were reduced after the treatment with Zuogui. **(C)** The levels of DA and β-EP in PMS rats were enhanced after being treated with Zuogui. **(D)** PLS-DA analysis of metabolomics profiles for PMS rats treated with (green) and without Zuogui (red). **(E)** Metabolomic profiles of PMS rats (purple), PMS rats treated with Zuogui (blue), perimenopausal rats (green), and 6-month-old rats (red). “*” indicates the comparison with 6-month-old rats [*p* < 0.05 (*)]; “△” indicates the comparison with perimenopausal rats [*p* < 0.05 (△)]; “#” indicates the comparison with PMS rats [*p* < 0.05 (#)].

While Yougui is generally prescribed for kidney yang deficiency, the decoction of Zuogui (Zuogui) (Table 3) is for kidney yin deficiency in centuries of TCM practice (He et al., 2014). Treatment of PMS rats with Zuogui showed minimal influence on the body weight (Supplementary Figure S12). Similar to the treatment with Yougui, treatment with Zuogui did not lead to a significant change in the level of E2 in PMS rats (Figure 5A), but the levels of CORT and ACTH were reduced, and the level of DA was increased. Unlike Yougui, Zuogui treatment increased the level of β-EP (Figures 5B,C).

Zuogui modulated the metabolomic state of the PMS rats (Figure 5D). Lipid, glucose, proteins, and trimethylamine-n-oxide were important metabolites influencing the metabolomic profile (Supplementary Figure S13). Although the metabolomic profile moved in the direction of the perimenopausal rats, the adjustment was not sufficient to bring the metabolomic state of PMS rats to that of 6-months old (Figure 5E).

Zuogui upregulated the levels of unsaturated lipid, glutamine, acetate, valine, and lipid while downregulated the levels of creatine, trimethylamine-n-oxide, creatinine, glutamine, pyruvate, acetoacetate, and glycoproteins in the PMS rats. Notably, after the Zuogui treatment, the levels of creatine, creatinine, glutamine, pyruvate, glycoproteins, and citrate were brought to the metabolic levels of the 6-month-old rats (Supplementary Figure S14 and Supplementary Table S4). It seemed that Zuogui attenuated the metabolism of PMS rats effectively, but it might also indicate that the PMS rats used here were more of kidney yin deficiency than kidney yang deficiency.

### Influence of CSS on the PMS Rats

CSS (Table 1) is a classical prescription of TCM to treat LQS-associated illness. CSS was documented as early as 1624 in a book of TCM, Jing Yue Quan Shu. The decoction is an effective prescription for ameliorating depression and anxiety (Su et al., 2011; Su et al., 2014).

**FIGURE 6:**
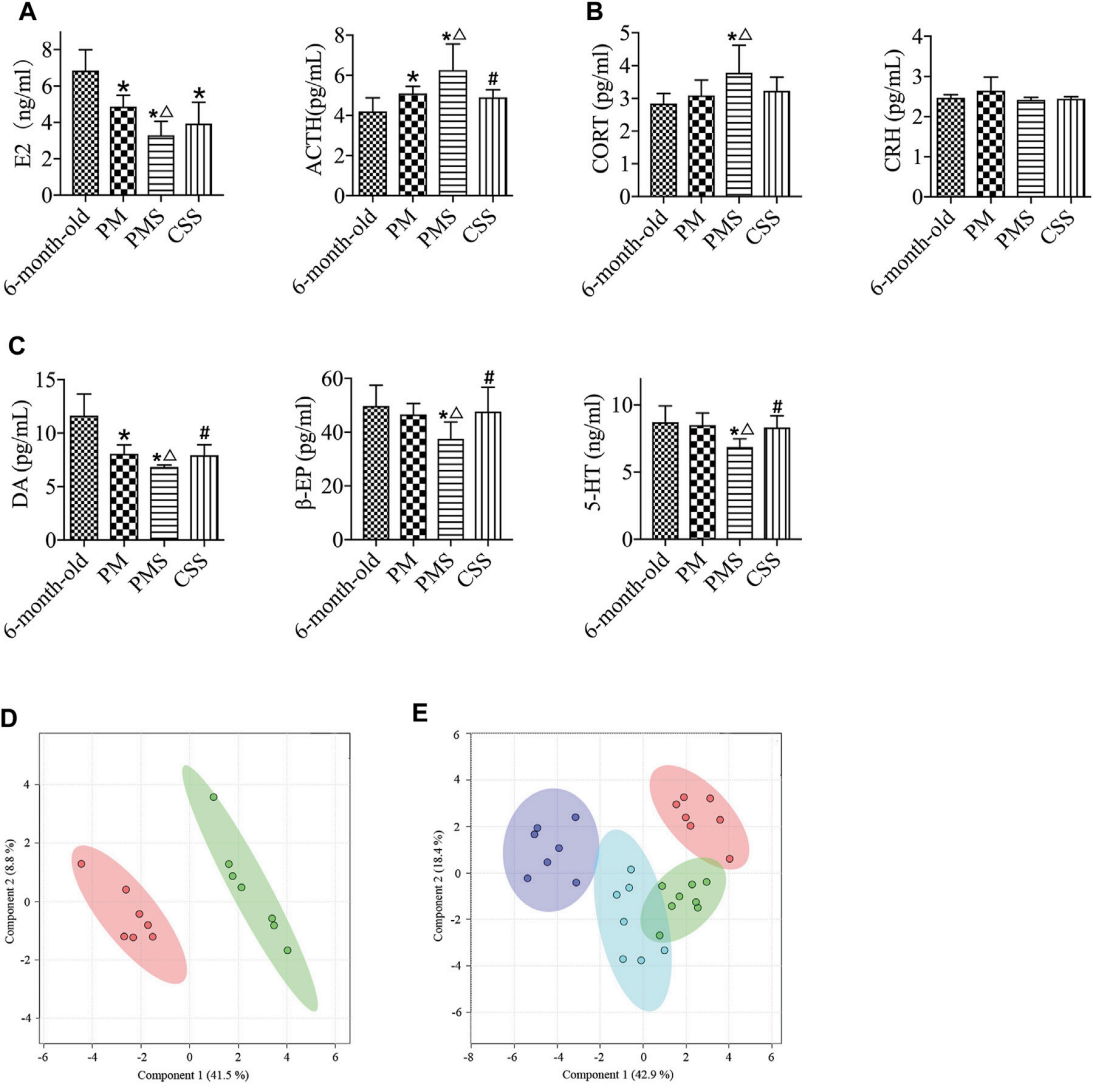
PMS rats treated with CSS. **(A)** The E2 level of the PMS rats with treatment was not significantly different. **(B)** Compared with the PMS rats, the levels of ACTH were reduced after treatment with CSS. **(C)**. The levels of β-EP, 5-HT, and DA in PMS rats were enhanced after the treatment with CSS. **(D)** Metabolomic profiles of PMS rats with (red) and without (green) the CSS treatment. **(E)** PLS-DA analysis of the metabolomics of plasma from that CSS-treated PMS (blue), PMS (purple), perimenopausal (green), and 6-month-old (red) rats. “*” indicates the comparison with 6-month-old rats [*p* < 0.05 (*)]; “△” indicates the comparison with perimenopausal rats [*p* < 0.05 (△)]; “#” indicates the comparison with untreated PMS rats [*p* < 0.05 (#)].

As shown in Supplementary Figure S15, not much change was observed in the weight of PMS rats treated with CSS as compared to that of the 6-month-old or perimenopausal rats. The changes in the levels of E2 and CORT were not significant either (Figures 6A,B). There was a significant reduction in the level of ACTH however and a considerable increase in the levels of β-EP, 5-HT, and DA in PMS rats treated with CSS, suggesting that CSS altered the endocrine parameters of PMS rats in the favorable direction (Figure 6C).

CSS treatment effectively modulated the metabolomic profile of PMS rats (Figure 6D). It brought the metabolomic state of the PMS rats closer to that of the PM rats but still not to that of the 6 months old (Figure 6E). It upregulated the levels of the unsaturated lipids, isoleucine, and lipid while downregulating the levels of glucose, creatine, betaine, creatinine, glutamine, pyruvate, and acetoacetate. The dominant metabolites for changing the metabolomic state by CSS were glucose and lipid (Supplementary Figure S16). In comparison with the 6-month-old rats, the levels of most of the metabolites were higher, including trimethylamine-n-oxide, betaine, choline, citrate, glutamine, acetoacetate, acetone, methionine, glycoprotein, acetate, valine, isoleucine, leucine, and lipid. Moreover, the levels of glucose, creatine, pyruvate, glycerol, creatinine, and succinate were adjusted to that of the 6 months old Supplementary Figure S17, Supplementary Table S5). Overall, CSS upregulated the metabolism of lipid and downregulated the metabolism of glucose. Considering that LQS rats were with upregulated glucose metabolism and downregulated lipid metabolism, the data is consistent with the fact that CSS is with a tranquilizing potency.

### A Combination Treatment for PMS

The aforementioned data indicated that both glucose and lipid metabolisms were upregulated in perimenopausal rats. In LQS rats, however, the glucose metabolism was further upregulated, while the lipid metabolism was downregulated. It was previously shown that the glucose and lipid metabolisms were both upregulated for the perimenopausal rats by the treatment with either Yougui or Zuogui (Chen et al., 2020). For the PMS rats though the glucose metabolism was downregulated, the lipid metabolism was upregulated for PMS rats treated with either Yougui, Zuogui, or CSS. It seemed that each of these decoctions was of various degrees of benefits in treating PMS rats based on the metabolomic indicators. However, none of these decoctions was effective enough to bring the metabolomic state of PMS rats to that of rats before entering the perimenopause.

As different decoctions for KD and LQS seem to adjust the metabolomics in different directions and attenuate different sets of metabolites, one would naturally wonder what would be the effect if both KD and LQS were ameliorated simultaneously. To this end, a decoction containing ingredients from Zuogui-CSS was prepared for the gavage of PMS rats.

The weight of PMS rats was not dramatically changed by the treatment with this decoction containing ingredients from Zuogui-CSS (Supplementary Figure S18A). The level of E2 was not significantly altered with the treatment either (Supplementary Figure S18B). The ACTH and CORT levels of peripheral blood dropped after the treatment, but the levels of DA, β-EP, and 5-HT increased (Supplementary Figures S18C,D).

**FIGURE 7:**
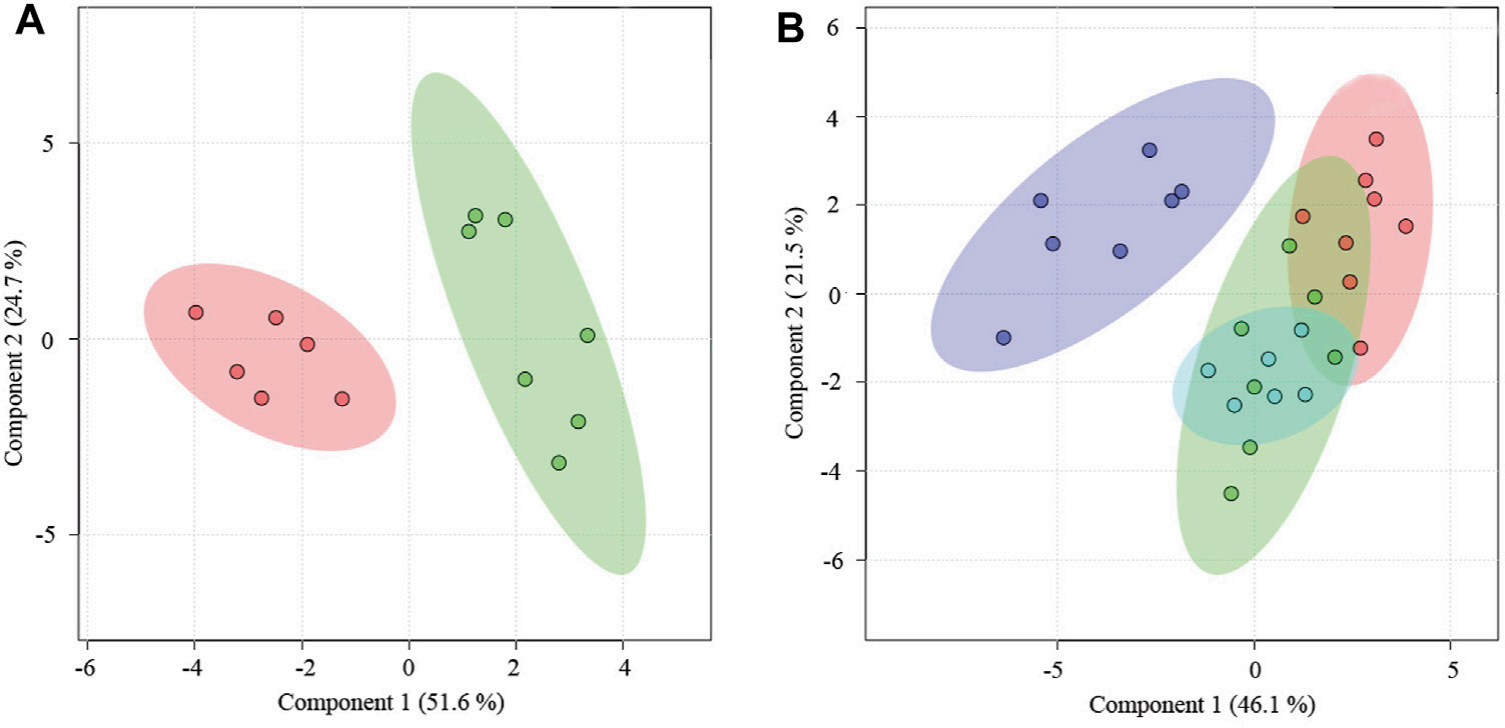
A combination treatment for PMS. **(A)** PLS-DA analysis of metabolomics for the plasmas of PMS rats treated with (red) and without Zuogui-CSS (green). **(B)** Metabolomic profiles of the plasmas of PMS rats treated with Zuogui-CSS (blue), PMS rats (purple), perimenopausal rats (green), and rats of 6 months old (red).

The metabolomic analysis indicated that the combined treatment of Zuogui-CSS was effective for restoring a metabolic state of PMS (Figure 7). Treatment with Zuogui-CSS not only adjusted the metabolomic state of the PMS rats to that of the perimenopausal rats but also brought the metabolomic profile of PMS rats to the proximity of the metabolomic profile of the 6-month-old rats (Figure 7B). Further analysis indicated that Zuogui-CSS modulation of the metabolome of PMS rats was with lipid and glucose as the dominant factors (Supplementary Figure S19). The Zuogui-CSS treatment significantly lowered the levels of glucose, glycerol, trimethylamine-n-oxide, betaine, glutamine, acetoacetate, methionine, glycoprotein, valine, and leucine, but the levels were still higher than those of the 6-month-old rats. In contrast, the pyruvate concentration in PMS rats was even lower than that in the 6-month-old rats after the treatment. Other metabolites, such as creatine, citrate, succinate, acetoacetate, acetone, glycine, and isoleucine, were of similar levels between PMS rats treated with Zuogui-CSS and the 6-month-old rats (Supplementary Figure S20; Supplementary Table S6).

### No Detectable Estrogenic Compounds in the Decoction for Treating PMS

**FIGURE 8:**
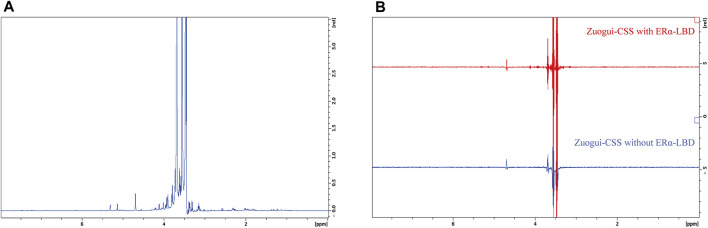
STD NMR detection for estrogenic compounds in Zuogui-CSS. **(A)** The 1D ^1^H NMR spectrum of Zuogui-CSS. **(B)** The STD spectra of Zuogui-CSS in the presence of ERα-LBD (upper red spectrum) and in the absence of ERα-LBD (lower blue spectrum). Only nonspecific solvent signals from glycerol (3.54, 3.63, and 3.77 ppm) and H_2_O (4.70 ppm) were observed in the STD spectra.

It was possible that the effective attenuation of the metabolomic state of PMS rats without significant change in the level of estrogen was through modulating the activity of estrogen receptors with interacting compounds in Zuogui-CSS. To address this possibility, the ligand-binding domain of estrogen receptor α (ERα LBD) was expressed and purified (Supplementary Figure S21). STD, a ligand-based NMR technique (Mayer and Meyer, 2001), was employed to detect the possible interacting molecules in the decoction. The STD experiment involves subtracting a spectrum, in which the protein was selectively saturated with magnetic radiation, from another spectrum without the protein saturation. In the difference spectrum, only the signals of those compounds that received the magnetic radiation transferred from the protein will remain. Other compounds that do not bind to the protein will not receive the saturation transfer so their characteristic signal will not appear in the difference spectrum (Meyer and Peters, 2003). The techniques can detect K_d_ values ranging between 10^–3^ and 10^–8^ M for the interacting compounds and is especially useful for detecting weak binders (Mayer and Meyer, 2001). Many phytoestrogens are with affinities in this range to estrogen receptors. For example, the K_d_ value for daidzein binding ERα is 5.9 × 10^−6^ M (van Lipzig et al., 2004) and that for coumestrol is 1 × 10^−4^ M (Qiu et al., 2020). So the technique is applicable to detecting the like compounds in the decoctions if they are of reasonable concentration. The spectrum without the saturation transfer from the protein was obtained for Zuogui-CSS (Figure 8A). In the STD difference spectrum (the upper spectrum in Figure 8B), the only observed peaks were those from glycerol (3.54, 3.63, and 3.77 ppm) and H_2_O (4.70 ppm) in the solution (Lu et al., 2018). These were nonspecific peaks due to their high concentrations as they also were present in the spectrum without the protein (lower spectrum in Figure 8B). So, there was no detectable compound by STD in the decoction to interact with ERα.

**FIGURE 9:**
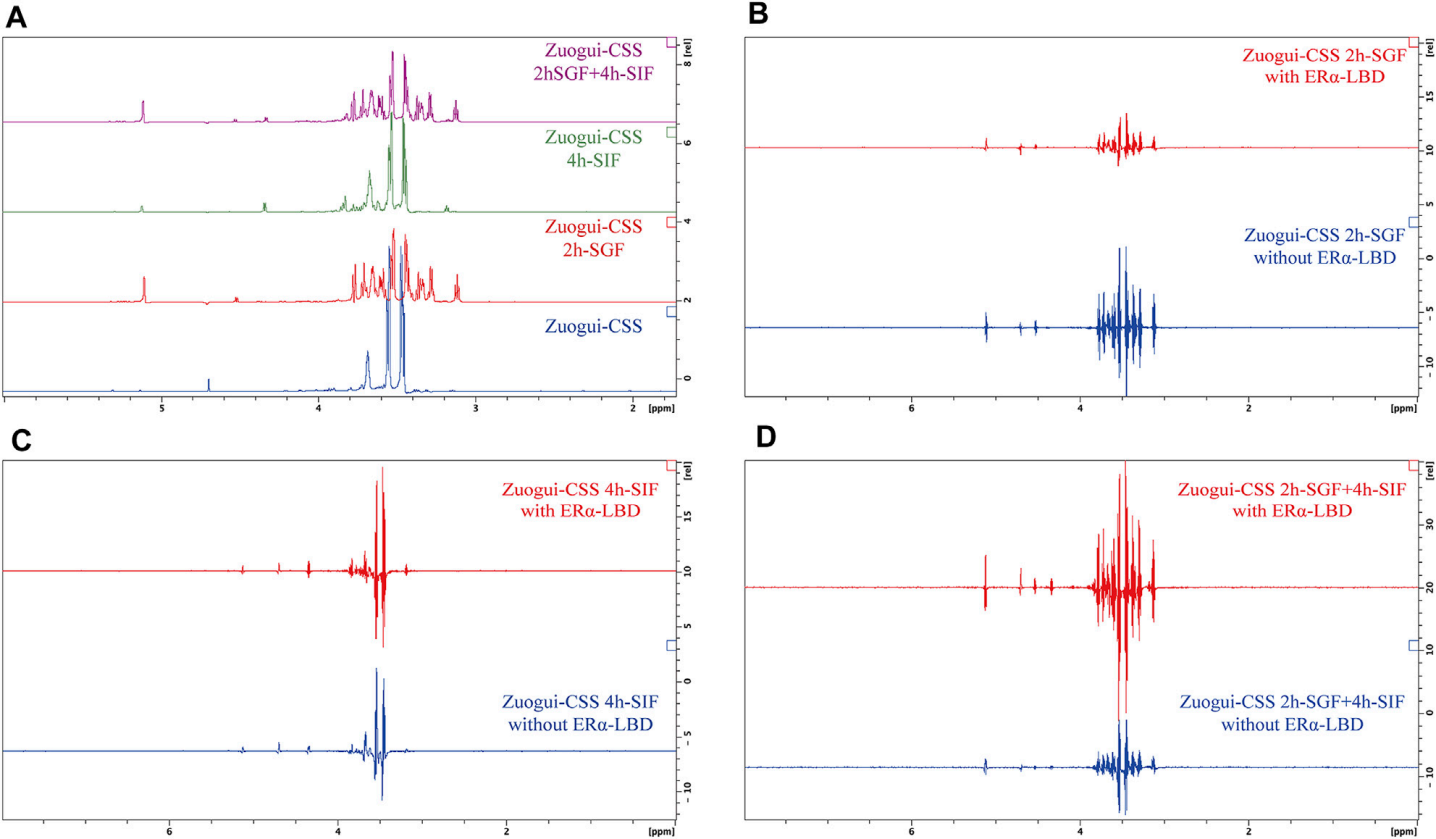
Gastrointestinal digestion of Zuogui-CSS produced no estrogenic compound. **(A)** ^1^H NMR spectra of Zuogui-CSS treated with simulated gastrointestinal fluids. The blue-colored spectrum was for Zuogui-CSS without the treatment. The green-colored spectrum was for Zuogui-CSS treated with SIF for 4 h. The red-colored spectrum was for Zuogui-CSS treated with SGF for 2 h. The purple-colored spectrum was for Zuogui-CSS sequentially treated with SGF for 2 h and SIF for 4 h. Peaks for new compounds appeared after Zuogui-CSS was treated with either SGF, SIF, or both. **(B)** The STD spectra of Zuogui-CSS treated with SGF for 2 h. The upper red-colored spectrum was from the sample with ERα-LBD and the lower blue-colored spectrum was from the sample without ERα-LBD. **(C)** The STD spectra of Zuogui-CSS treated with SIF for 4 h. The upper red-colored spectrum was from the sample with ERα-LBD and the lower blue-colored spectrum was from the sample without ERα-LBD. **(D)** The spectra of Zuogui-CSS were treated sequentially with SGF for 2 h and SIF for 4 h. The upper red-colored spectrum was from the sample with ERα-LBD and the lower blue-colored spectrum was from the sample without ERα-LBD. The STD spectra were with only solvent signal peaks (e.g., glycerol, H_2_O, and self-degradation products of pepsin or pancreatin). There was no indication of any compound that could interact with ERα-LBD.

As these decoctions were through the stomach and intestinal tracts after the gavage, the gastrointestinal digestion could yield interacting compounds. To test this possibility, Zuogui-CSS was treated with SGF and SIF for the STD NMR experiments. The treatment with either SGF or SIF indeed produced new compounds (Figure 9A). The decoction was also first treated with SGF and then with SIF simulating its passage through the gastrointestinal system. The resulting spectrum for the sequential treatment of Zuogui-CSS though was the superposition of spectra from Zuogui-CSS treated with SGF and SIF separately (Figure 9A). These newly generated compounds by the gastrointestinal digestion were with no detectable interaction with ERα LBD either based on the STD NMR analyses (Figures 9B–D).

## Discussion

Perimenopause is a period of transition to menopause characterized by irregularity in the menstrual cycle with fluctuating and overall declining levels of estrogen in women. As the body conditions going through a significant transformation with the gradual cessation of reproductive functions, many perimenopausal women suffer from PMS. Hormone replacement therapy (HRT) is an established clinical practice for treating PMS (Whiteley et al., 2013). However, serious side effects have also been reported for HRT (Hickey et al., 2005; Sare et al., 2008). For a transition to menopause, the declining level of estrogen and reproductive senescence is a natural process of aging. Supplementation of estrogenic compounds, such as that in HRT, could be countering a natural trend in the body. By directly compensating for the loss of estrogen, the PMS symptoms might be soothed but it could also be counterproductive and result in undesirable side effects. A better treatment strategy might be to palliate the symptoms of PMS without interfering with the body’s natural transition to menopause such as supplementing estrogenic compounds.

The association of PMS with KD and LQS in TCM seems enigmatic and jargonistic to the general practitioners of medicine. However, the fact that the subsequent prescriptions based on the theory of TCM are effective is an indication that it is with sound rationales. Our data here indicated that Zuogui, Yougui, and CSS were efficacious to various degrees for treating PMS, with Zuogui-CSS adjusting body’s conditions to a more seemingly desirable direction in a PMS model that was deemed to be with both KD and LQS. The best strategy, however, seemed to be treating both KD and LQS simultaneously with a combined decoction of Zuogui and CSS for the PMS rats. This combination treatment could bring the metabolomic state of PMS close to that of rats at a younger age before entering perimenopause. It was even more remarkable that this amelioration was done seemingly neither by directly upregulating the estrogen level nor by supplementing an estrogenic compound.

The dose of phytoestrogens for treating PMS is in the range of hundreds of milligrams to grams for humans (Crisafulli et al., 2005; Xiao, 2008), which would be about milligrams to hundreds of milligrams for rats considering the difference in size and HED. Taking into accounts the volume of decoction feeding to the animal (4–5 ml) and the averaged molecular weight of phytoestrogens (∼250), the useful concentration for phytoestrogens should be around a few millimolars to hundreds of millimolars. Since the STD technique employed here is capable of detecting interacting compounds in the range of low millimolars to nanomolars, or even lower, the detection of no interacting compound in Zuogui-CSS by STD indicates that either there was just no such compound or the amount of the compound was too small to effectively modulate the activity of estrogen receptors like those estrogenic compounds did in HRT. In light of the fact that the ingredients from two decoctions were to be mixed to yield the desirable results, it is possible that multiple factors are in play to attenuate the metabolomic state of the PMS model.

The doses used for this study seem to be an important factor to obtain the results in this study as a previous study showed that the increased dosage of Zuogui at daily applications of either 13.78 g/kg, 20.67 g/kg, or 31 g/kg for each rat, as compared to the daily dose of 3.04 g/kg for each rat in this study, could lead to the increased serum level of estrogen in a dose-dependent manner (Zhao et al., 2011). It implies that a well-calibrated and moderate dose of botanical drugs, Zuogui in this particular case, is important for ameliorating the disorder while avoiding the side effects as it is desirable to treat PMS without elevating the level of estrogen to counter the natural trend of the body’s transition to menopause.

The consistency between the metabolomic data in this study and the time-honored clinical practice of the abovementioned traditional Chinese prescriptions is an indication that the metabolomic indicators are applicable for assessing the efficacy of treating PMS with Chinese botanical drugs. It also implicates metabolomics as an invaluable tool to guide the use of traditional Chinese botanical drugs, in this case, by using a lower dose without elevating the level of serum estrogen for treating the disorder.

As effective as the treatment of the PMS model seems to be with the current prescription, the molecular mechanism of the treatment awaits further investigation. Does a ligand-independent ER pathway form the molecular basis of the TCM treatment of PMS? Or does the medicine applied here activate an ER-independent signaling pathway? In either case, the data presented here afford solid evidence for a better strategy to treat PMS with the traditional Chinese prescription of botanical drugs.

## Data Availability

The original contributions presented in the study are included in the article/Supplementary Material; further inquiries can be directed to the corresponding authors.

## Abbreviations

CSS, Chaihu-Shugan-San; ELISA, enzyme-linked immunosorbent assay; 1H NMR, proton nuclear magnetic resonance; HRT, hormone replacement therapy; KD, kidney deficiency; LQS, liver qi stagnation; OPLS-DA, orthogonal partial least-squares-discriminant analysis; PMS, perimenopausal syndrome; SPF, specific pathogen free; STD, saturation transfer difference; TCM, traditional Chinese medicine; VIP, variable influence on projection.
