# Supplementary material for: An Effective Treatment of Perimenopausal Syndrome by Combining Two Traditional Prescriptions of Chinese Botanical Drugs
Source: Front Pharmacol. 2021 Oct 25;12:744409. doi: 10.3389/fphar.2021.744409 (PMC8573068; doi:10.3389/fphar.2021.744409)
Supplement: Supplementary file 1 [file DataSheet1.PDF]

Supplementary Materials

to

A combination treatment of perimenopausal syndrome with Chinese herbs is metabolomically restorative

by

Junjie Lan[1†], Caiming Wu[1†], Wen'na Liang[2], Jianying Shen[2], Zewei Zhuo[2], Liu Hu[2], Luwei Ruan[2], Pengheng Zhang[2], Xiangrong Ye[3], Leqin Xu[3], Chengfu Li[3], Shengyuan Lin[4], Chuanhui Yang[1], Siqi Wu[1], Yingjun Dong[1], Haixia Ren[1], Huiying Huang[1], Bizhen Gao[2], Hongwei Yao[5], Tianwei Lin[1], Xueqin Chen[3,6*] and Candong Li[2*]

Supplementary Figure 1.

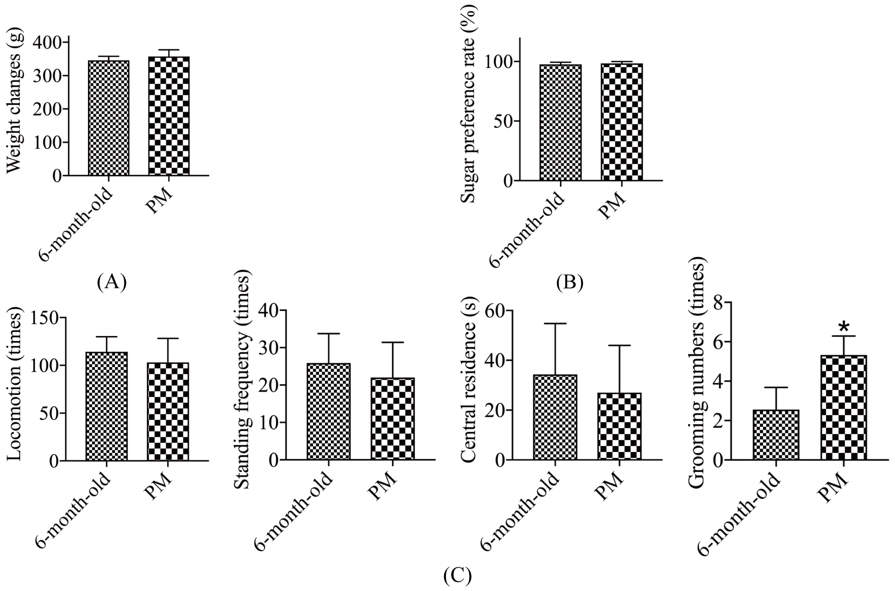

Weight, sugar preference and physical activities of perimenopausal rats. (A) The body weight and (B) sugar preference were of no significant difference between the 6-month-old and perimenopausal rats. (C) Behavior of the rats was recorded and scored according to the time spent in central squares, locomotion, frequency of standing, and number of grooming. Only the number of grooming showed statistical significance between the 6-month-old and perimenopausal rats. "*" indicates the comparison with 6-month-old rats (p<0.05 (*)).

Supplementary Figure 2

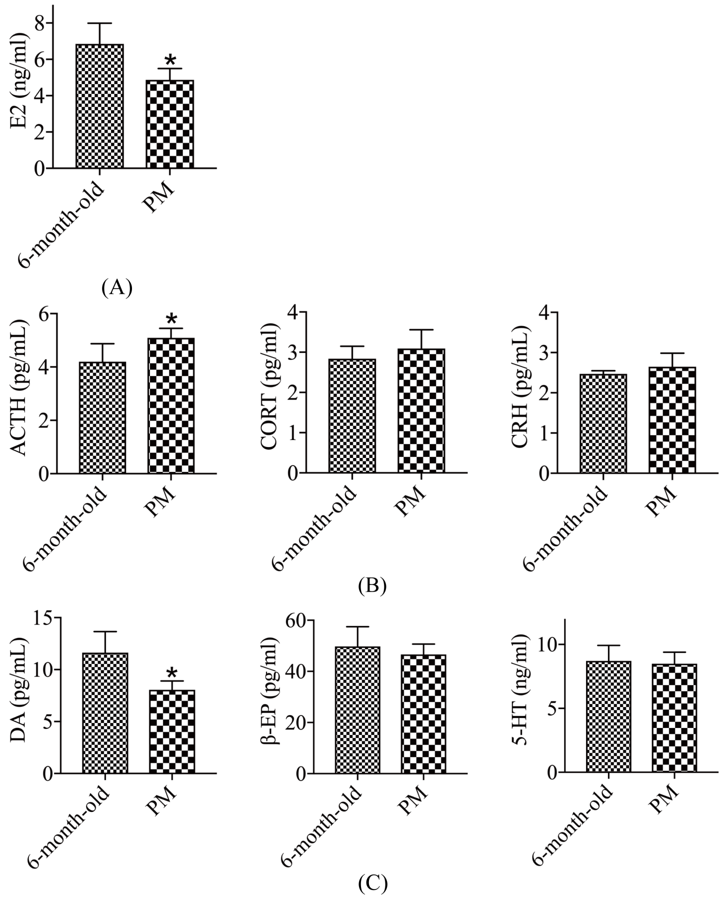

Estrogen, endocrines and neural transmitters in the experimental rats. (A) The level of estrogen was lower in perimenopausal rats. (B) Levels of CRH, ACTH and CORT. Compared with the 6-month-old rats, the levels of ACTH were enhanced. (C) Levels of β-EP, 5-HT and DA. Compared with the 6-month-old rats, the levels of DA in perimenopausal rats were lower. "*" indicates the comparison with 6-month-old rats (p<0.05 (*))

Supplementary Figure 3

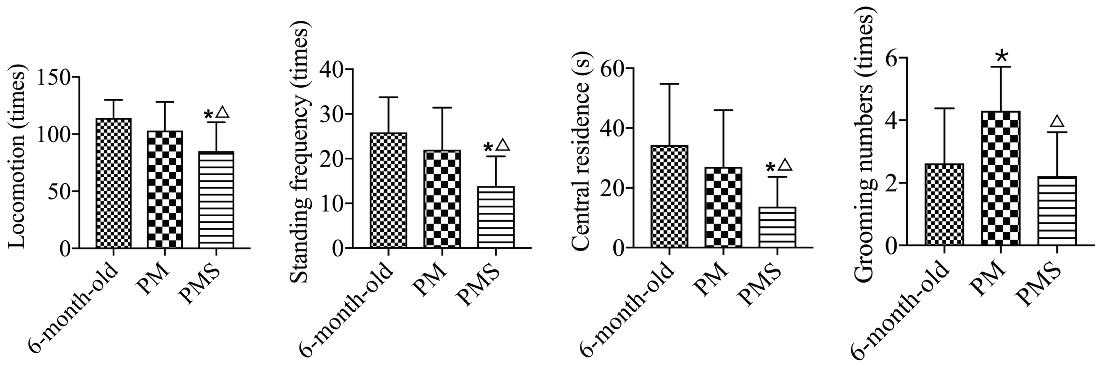

The rat behaviors were recorded and scored according to the time spent in central squares, locomotion, frequency of standing, and number of grooming. PMS rats had a reduced level of exercise compared with the 6-month-old rats and PM rats. "*" indicates the comparison with the 6-month-old rats ($p<0.05$ (*)); "△" indicates the comparison with the perimenopausal rats ($p<0.05$ (△)).

Supplementary Figure 4

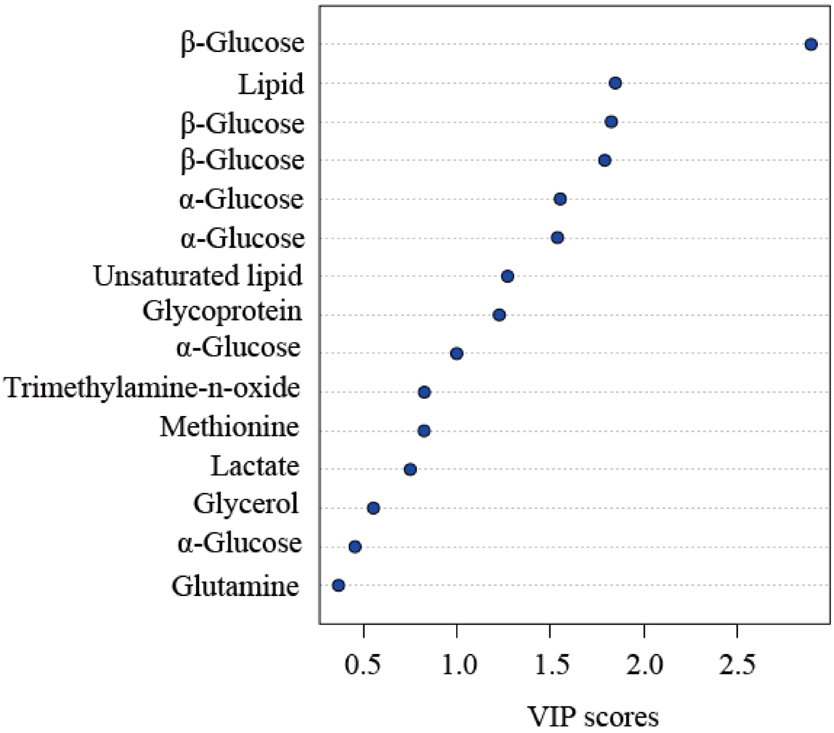

In the process of becoming LQS, glucose and lipid were the leading metabolites that changed the metabolomic state.

Supplementary Figure 5

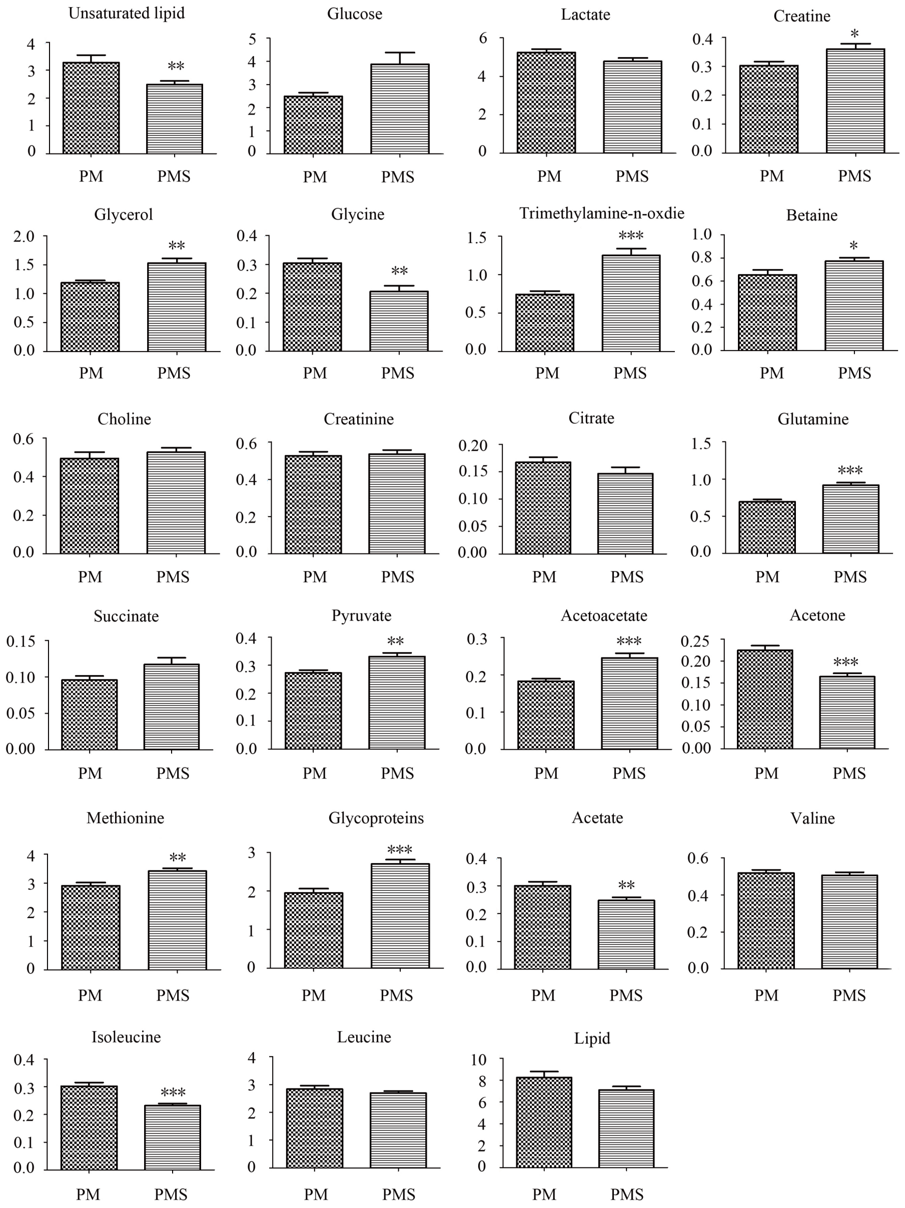

The metabolomic state of the rats changed significantly after the PM rats were subjected to CRS. Glucose metabolism was up-regulated, lipid metabolism was inhibited. Overall, the concentrations for unsaturated lipid, glycine, acetone, acetate, and isoleucine decreased, while the concentrations for glycerol, trimethylamine-n-oxide, betaine, glutamine, pyruvate, acetoacetate, methionine, and glycoprotein increased significantly. $p < 0.05$ (*); $p < 0.01$ (**); $p < 0.001$ (***).

Supplementary Table 1.

**Metabolites**	**PMS *vs* Perimenopausal rats**
Unsaturated lipid	↓**
Creatine	↑*
Glycerol	↑**
Glycine	↓**
Trimethylamine-n-oxide	↑***
Betaine	↑*
Glutamine	↑***
Pyruvate	↑**
Acetoacetate	↑***
Acetone	↓***
Methionine	↑**
Glycoproteins	↑***
Acetate	↓**
Isoleucine	↓***

↑ and ↓indicate either the increased or decreased metabolite concentrations in the plasma. $p<0.05$ (*); $p<0.01$ (**); $p<0.001$ (***).

Supplementary Figure 6

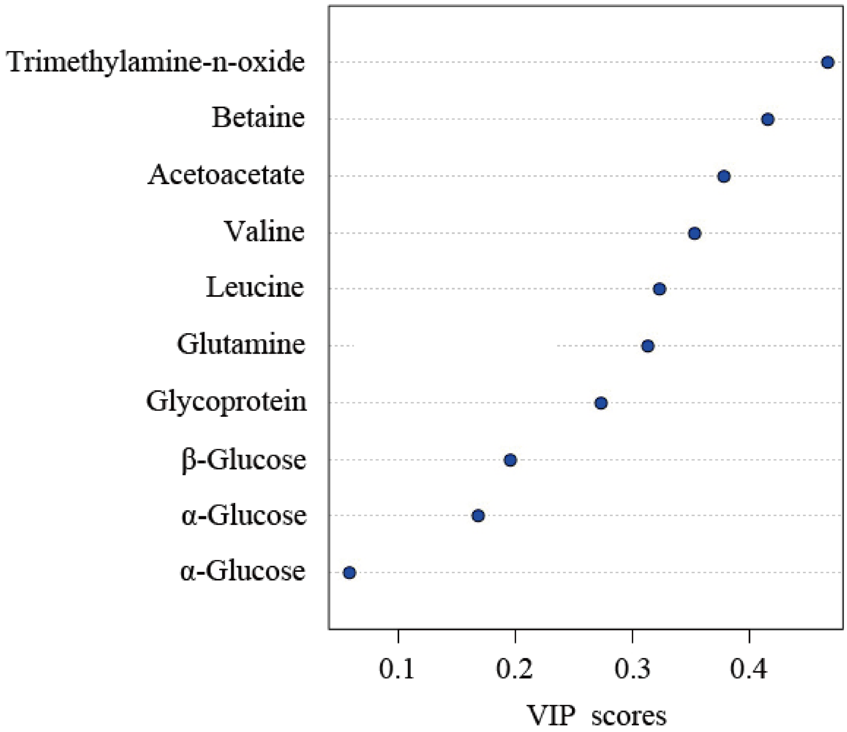

Compared with 6-month-old, the leading metabolites that changed the metabolomic state of PMS rats were trimethylamine-n-oxide and betaine.

Supplementary Figure 7

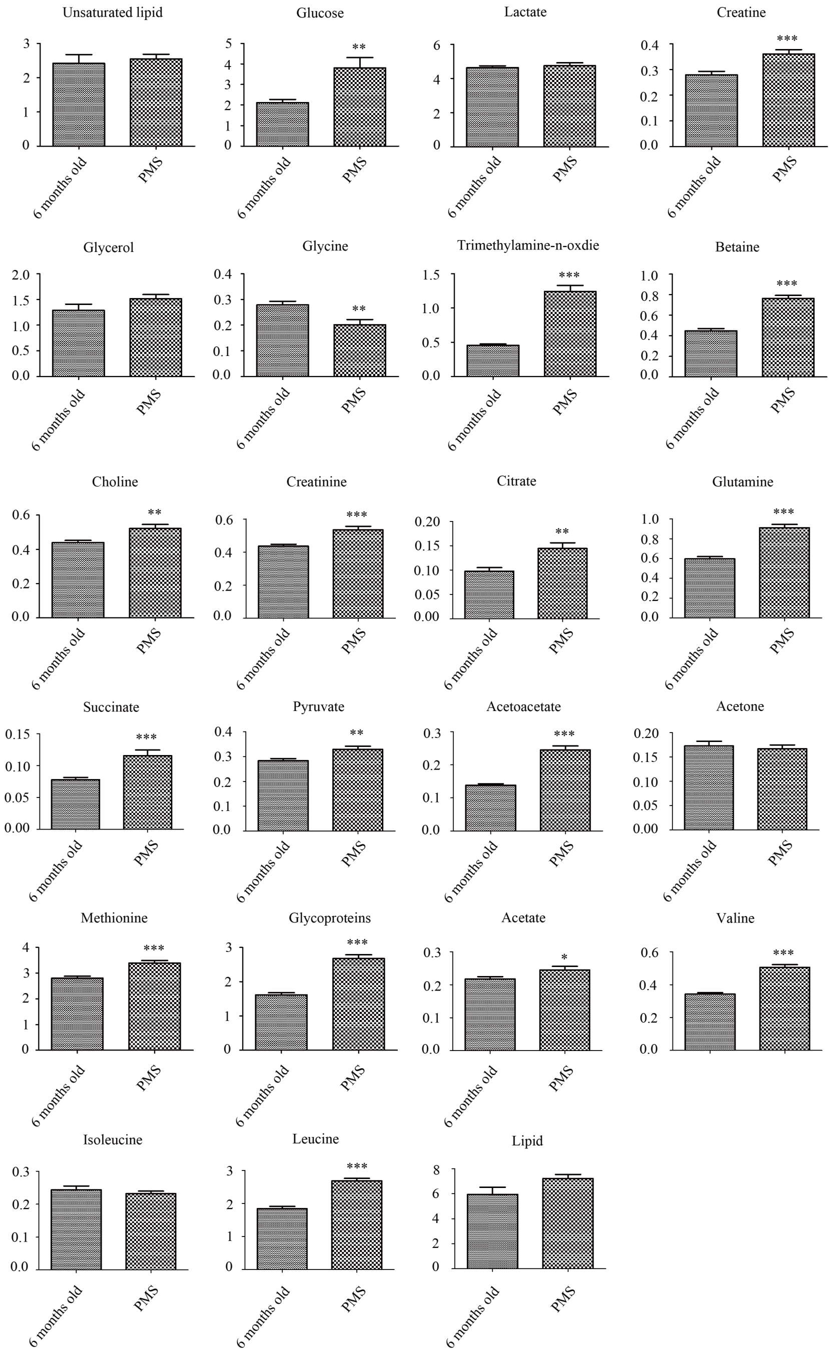

The metabolomics of the PMS rats changed significantly from the 6-month-old rats. Overall, the concentrations of glucose, creatine, trimethyl-amine N-oxide, betaine, choline, creatinine, citrate, glutamine, succinate, pyruvate, acetoacetate, methionine, glycoprotein, acetate, and valine increased significantly, while the concentration of glycine decreased. $p<0.05$ (*); $p<0.01$ (**); $p<0.001$ (***).

Supplementary Table 2

**Metabolites**	**PMS *vs* 6-month-old rats**
Glucose	↑**
Creatine	↑***
Glycine	↓**
Trimethylamine-n-oxide	↑***
Betaine	↑***
Choline	↑**
Creatinine	↑***
Citrate	↑**
Glutamine	↑***
Succinate	↑***
Pyruvate	↑**
Acetoacetate	↑***
Methionine	↑***
Glycoproteins	↑***
Acetate	↓*
Valine	↑***
Leucine	↑***

↑ and ↓indicate either the increased or decreased metabolite concentrations in the plasma. $p < 0.05$ (*); $p < 0.01$ (**); $p < 0.001$ (***).

Supplementary Figure 8.

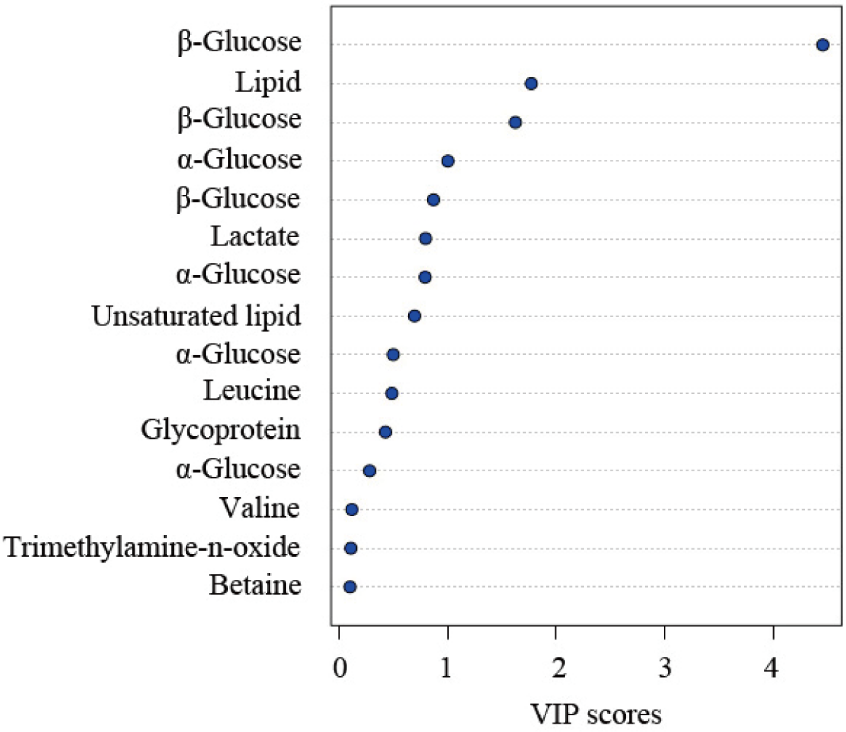

After treatment with Yougui, the dominant metabolites of PMS were all related to energy metabolism, with glucose and lipid at the top of the VIP list (VIP > 1: significant).

Supplementary Figure 9.

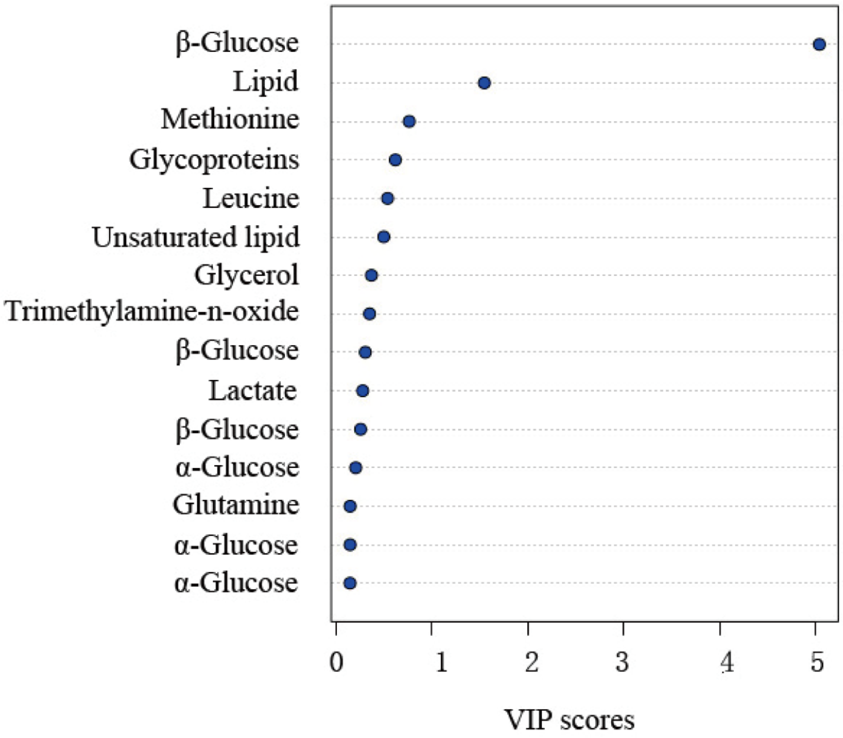

Compared with PM rats, the metabolism of glucose and lipid of PMS rats were modulated by Yougui, and at the top of the VIP list (VIP > 1: significant).

Supplementary Figure 10.

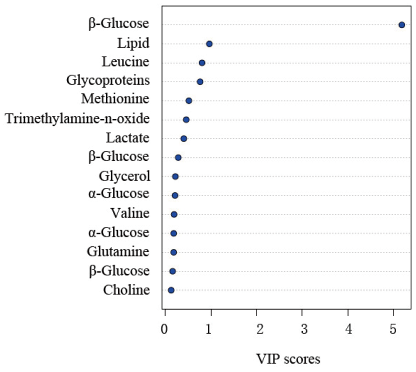

The glucose and lipid of PMS rats were at the top of the VIP list (VIP > 1: significant).

Supplementary Figure 11.

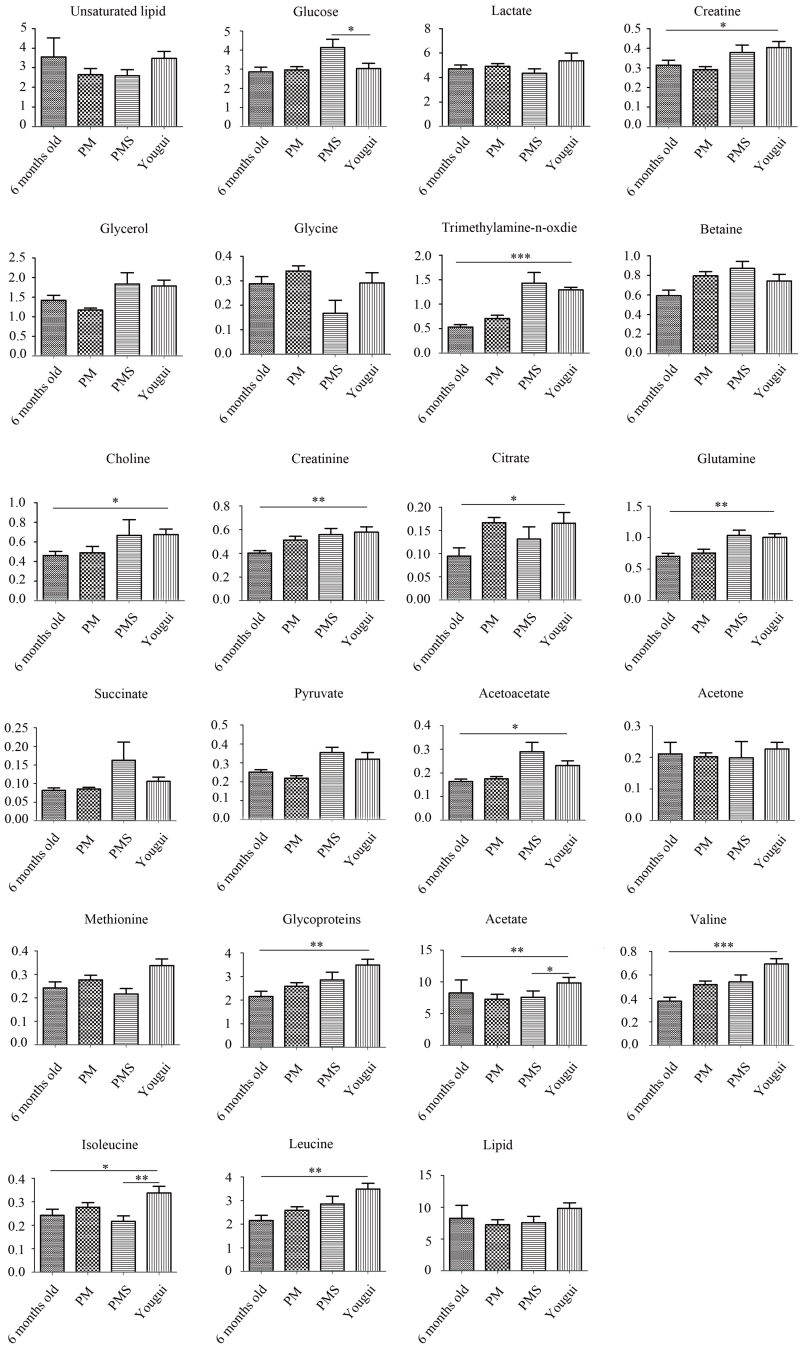

Yougui up-regulated the levels of acetate, isoleucine and down-regulated the level of glucose, but the level of trimethylamine-n-oxide was not significantly lower. Yougui treatment did not adjust the metabolomics of PMS rats to that of the 6-month-old rats. $p<0.05$ (*); $p<0.01$ (**); $p<0.001$ (***).

Supplementary Table 3.

metabolites	Yougui *vs* 6-month-old rats	Yougui *vs* PMS rats
Glucose		↓*
Creatine	↑*	
Trimethylamine-n-oxide	↑***	
Choline	↑*	
Creatinine	↑**	
Citrate	↑*	
Glutamine	↑**	
Acetoacetate	↑*	
Glycoproteins	↑**	
Acetate	↑**	↑*
Valine	↑***	
Isoleucine	↑*	↑**
Leucine	↑**	

↑and↓ indicate either the increased or decreased metabolite concentrations in the plasma.

$p<0.05$ (*); $p<0.01$ (**); $p<0.001$ (***).

Supplementary Figure 12.

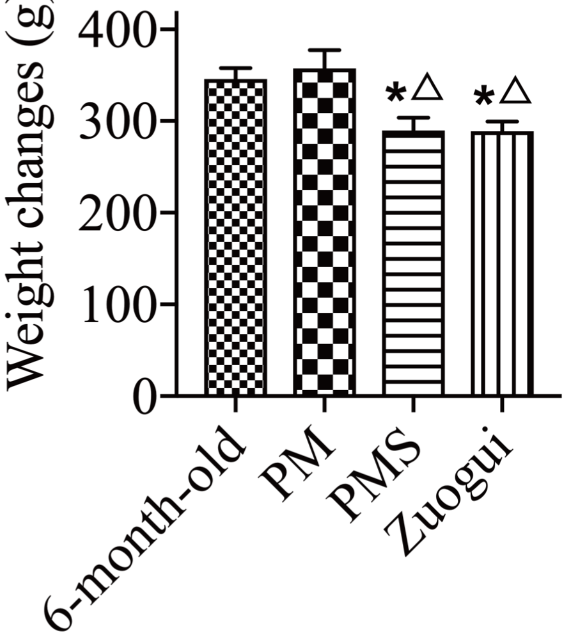

There was no statistically significant difference in the weight of PMS rats before and after treatment with Zuogui. "*" indicates the comparison with 6-month-old rats ($p<0.05$ (*)); "△" indicates the comparison with perimenopausal rats ($p<0.05$ (△)).

Supplementary Figure 13.

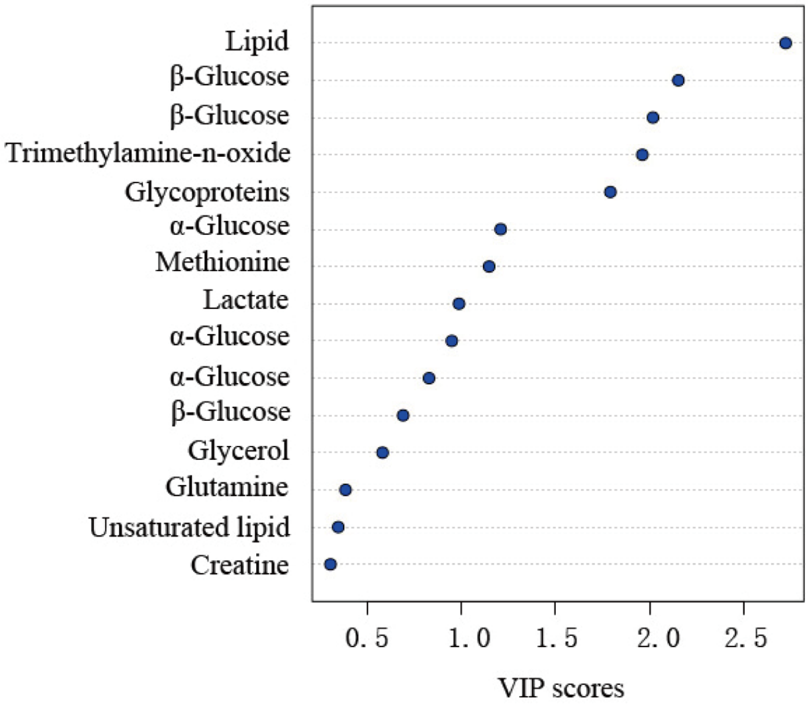

The metabolism of Lipid, glucose, trimethylamine-n-oxide, glycoproteins, methionine, and lactate of PMS rats were modulated by Zuogui. These metabolites were at the top of the VIP list (VIP > 1: significant).

Supplementary Figure 14.

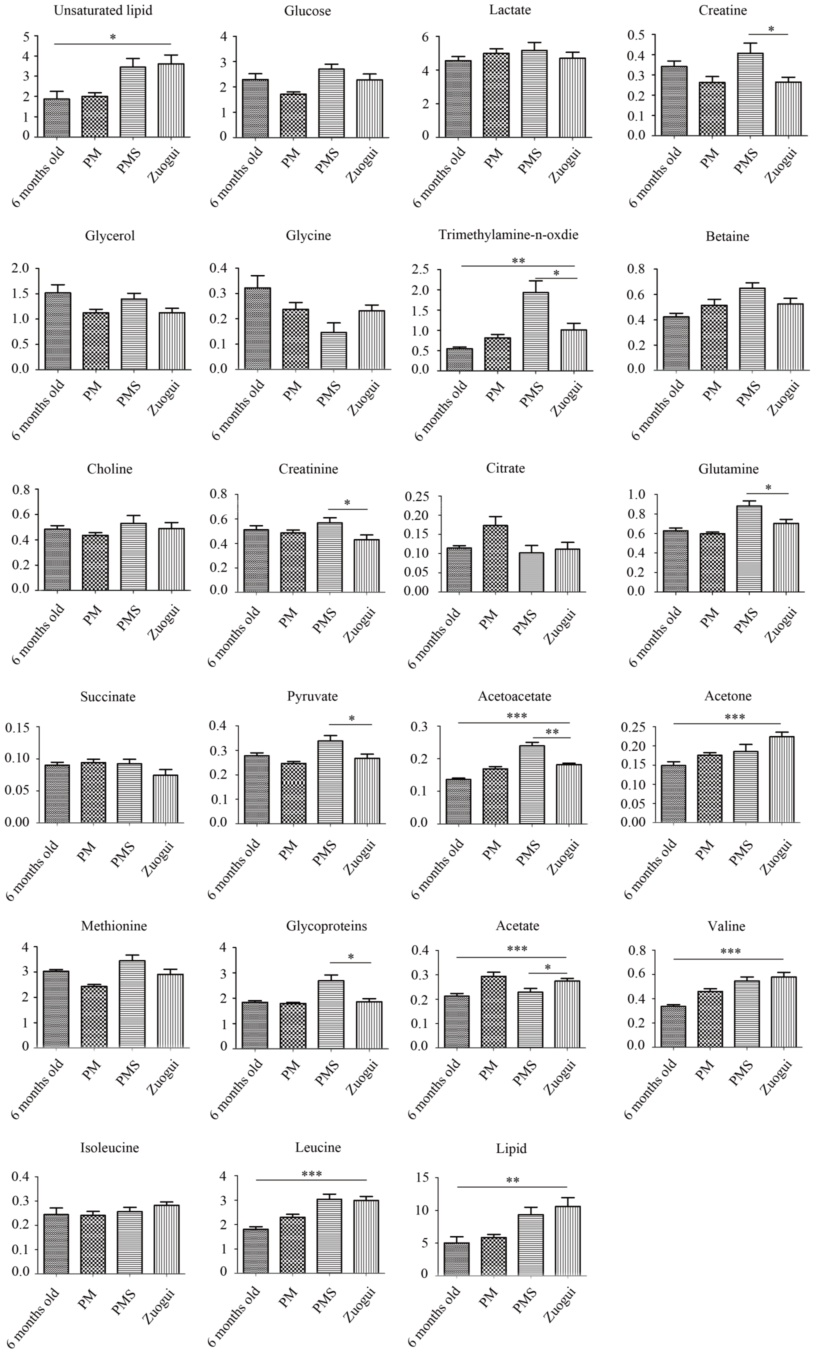

Zuogui treatment modulated the levels of creatine and creatinine to that of the 6-month-old rats. $p<0.05$ (*); $p<0.01$ (**); $p<0.001$ (***).

Supplementary Table 4.

metabolites	Zoogui vs 6-month-old rats	Zuogui vs PMS rats
Unsaturated lipid	↑*	
Creatine		↓*
Trimethylamine-n-oxide	↑**	↓*
Creatinine		↓*
Glutamine		↓*
Pyruvate		↓*
Acetoacetate	↑***	↓**
Acetone	↑***	
Glycoproteins		↓*
Acetate	↑***	↑*
Valine	↑***	
Leucine	↑***	
Lipid	↑**	

↑ and ↓ indicate either the increased or decreased metabolite concentrations in the plasma. $p < 0.05$ (*); $p < 0.01$ (**); $p < 0.001$ (***).

Supplementary Figure 15.

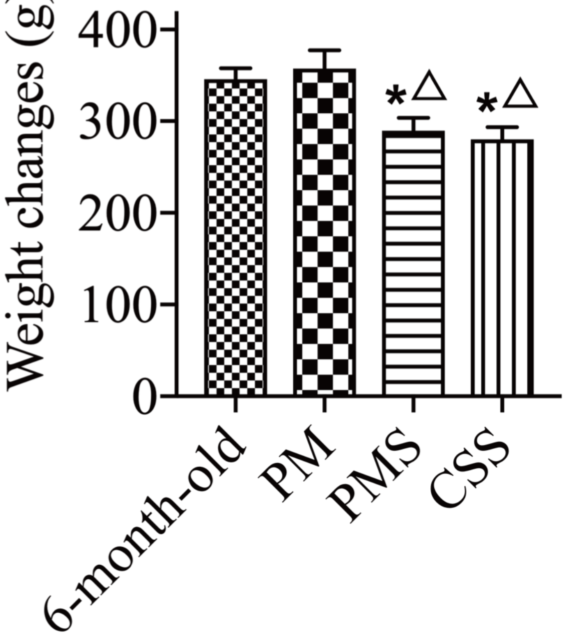

There was no statistically significant difference in the weight of PMS rats before and after treatment with CSS. “*” indicates the comparison with 6-month-old rats ($p<0.05$ (*)); “△” indicates the comparison with perimenopausal rats ($p<0.05$ (△)).

Supplementary Figure 16.

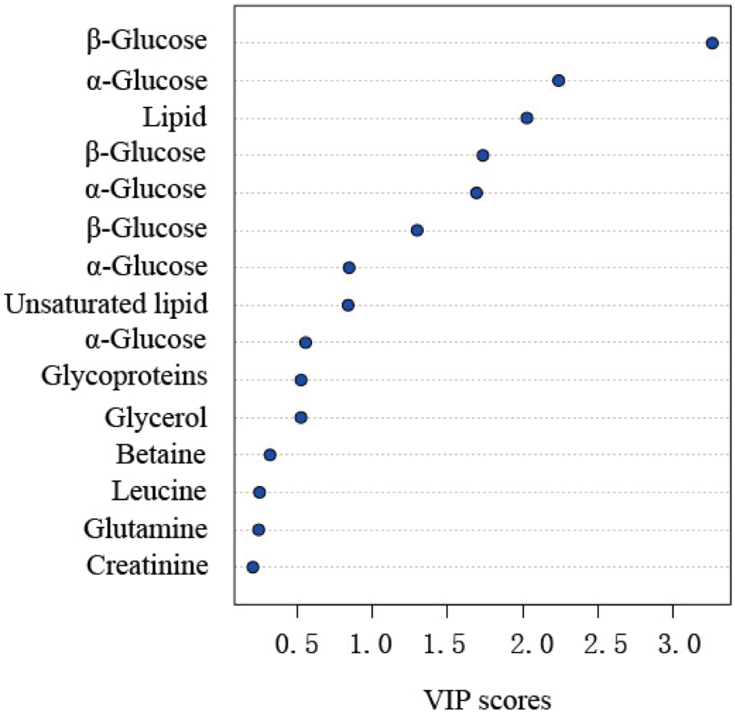

The metabolism of Lipid and glucose of PMS rats were modulated by CSS, which were at the top of the VIP list (VIP > 1: significant).

Supplementary Figure 17.

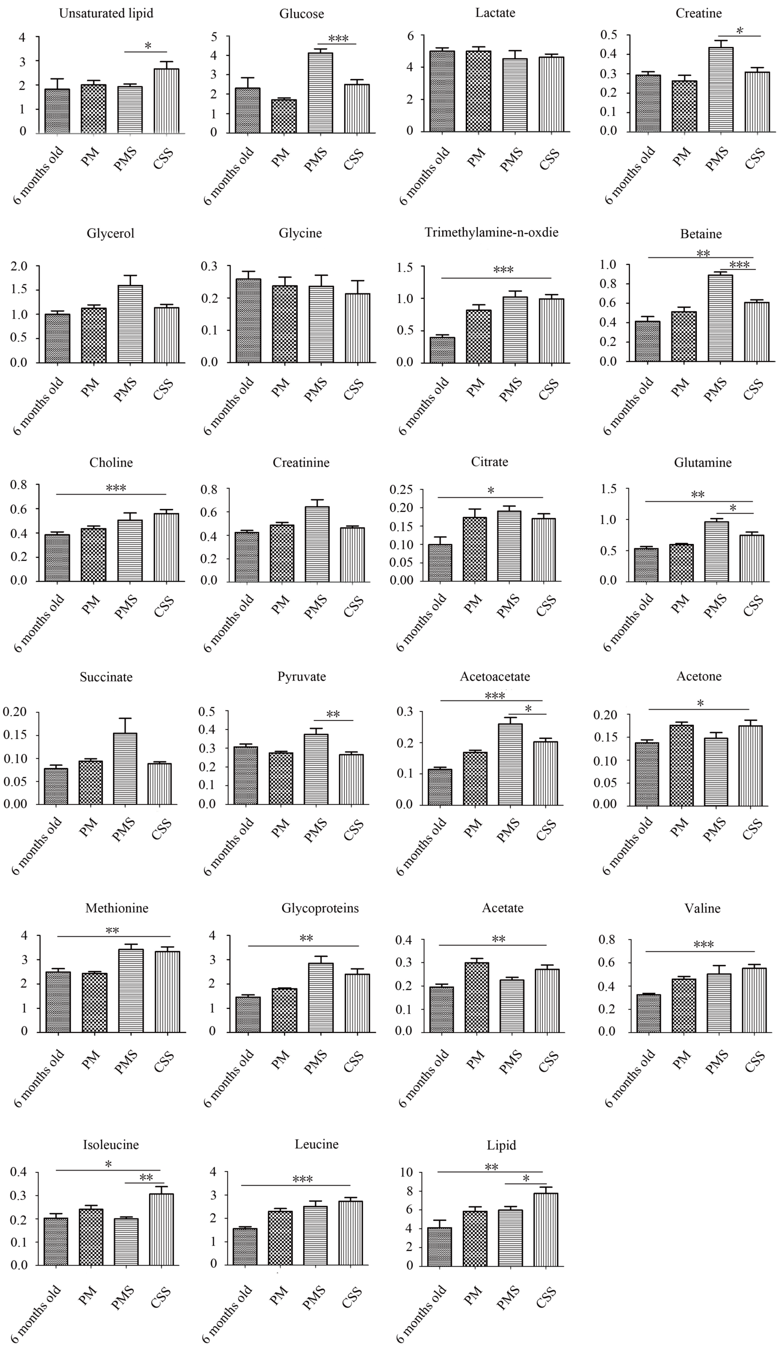

The treatment with CSS brought about a significant change in the metabolomic state of PMS rats. CSS treatment elevated the levels of the unsaturated lipids, isoleucine and lipid. The levels of glucose, creatine, glycerol, creatinine, succinate, and pyruvate were adjusted to those of the 6-month-old rats. However, other metabolites were still significantly different from those of 6-month-old rats. In general, CSS up-regulated the metabolism of lipid and down-regulated the metabolism of glucose. $p<0.05$ (*); $p<0.01$ (**); $p<0.001$ (***).

Supplementary Table 5.

metabolites	CSS *vs* 6-month-old rats	CSS *vs* PMS rats
Unsaturated lipid		↑*
Glucose		↓***
Creatine		↓*
Trimethylamine-n-oxide	↑***	
Betaine	↑**	↓***
Choline	↑***	
Creatinine		↓*
Citrate	↑*	
Glutamine	↑**	↓*
Pyruvate		↓**
Acetoacetate	↑***	↓*
Acetone	↑*	
Methionine	↑**	
Glycoproteins	↑**	
Acetate	↑**	
Valine	↑***	
Isoleucine	↑*	↑**
Leucine	↑***	
Lipid	↑**	↑*

↑ indicates the increase and ↓ indicates the decrease of metabolite concentrations in the plasma. $p<0.05$ (*); $p<0.01$ (**); $p<0.001$ (***).

Supplementary Figure 18.

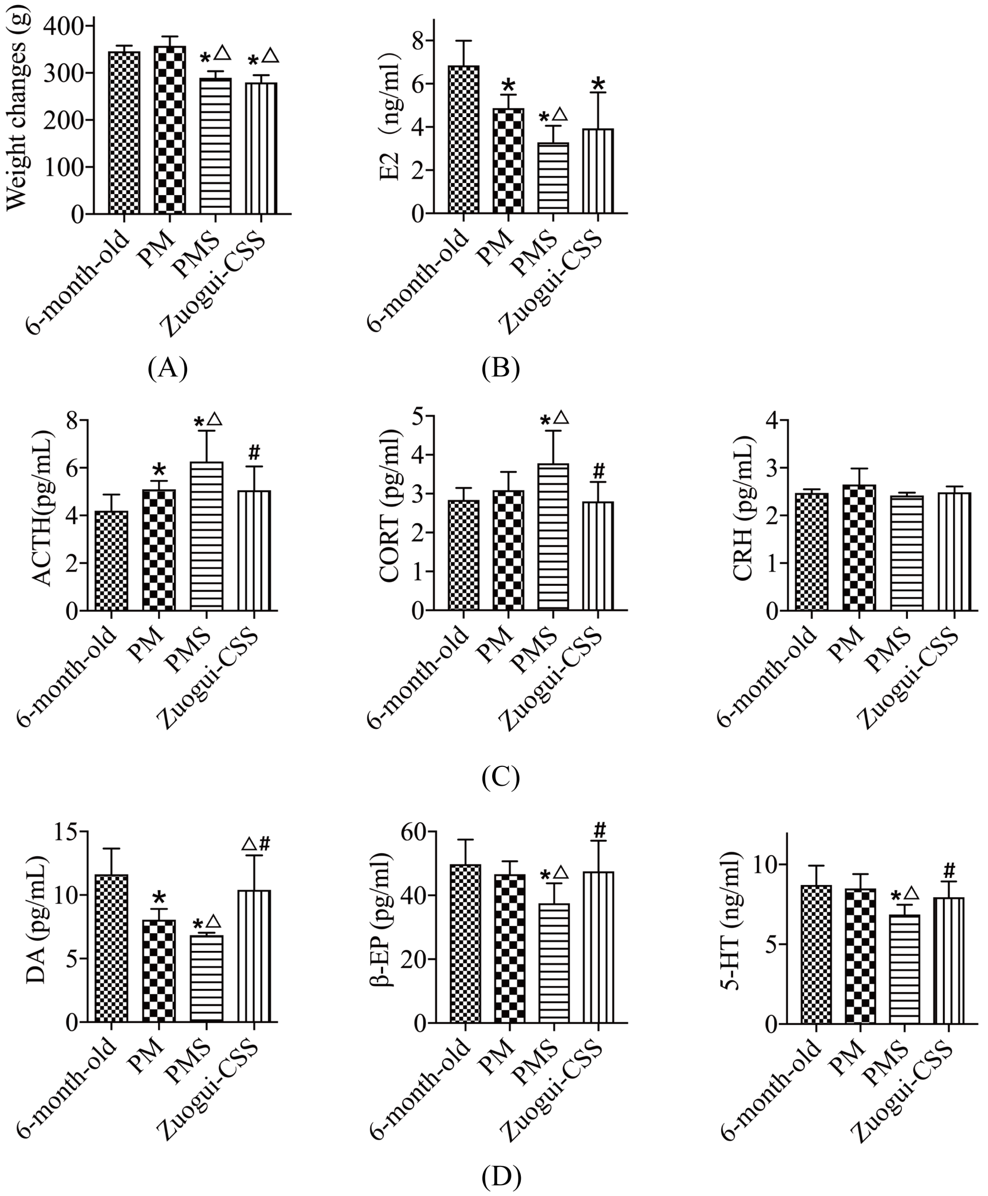

A combination treatment for PMS. (A) There was no statistically significant difference in the weight of PMS rats before and after treatment with Zuogui-CSS. (B) The E2 level of PMS rats was not significantly altered after treatment with Zuogui-CSS. (C) The levels of ACTH and CORT were reduced after being treated with Zuogui-CSS. (D) The levels of DA, β-EP and 5-HT in the PMS rats were enhanced after being treated with Zuogui-CSS. "*" indicates the comparison with 6-month-old rats ($p<0.05$ (*)); "Δ" indicates the comparison with perimenopausal rats ($p<0.05$ (Δ)); "#" indicates the comparison with PMS rats ($p<0.05$ (#)).

Supplementary Figure 19.

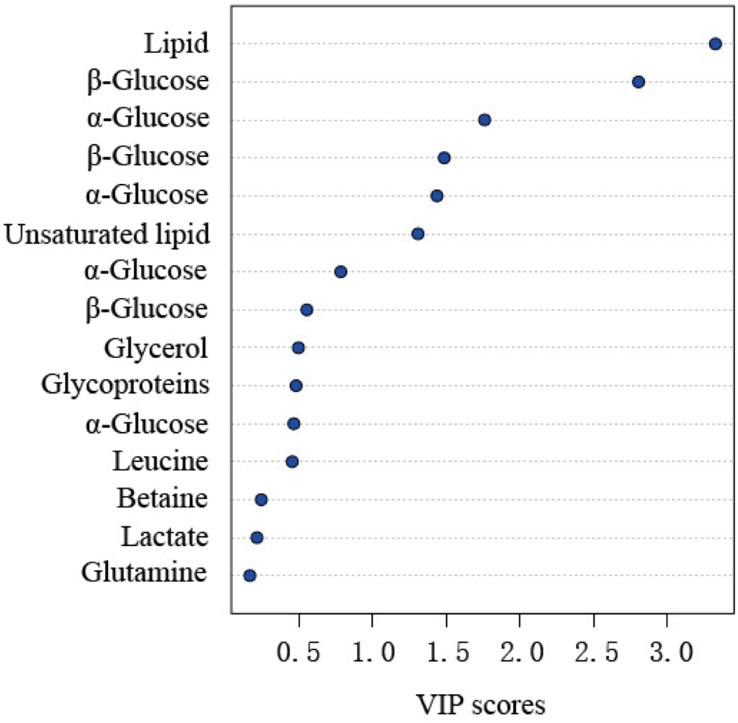

The metabolism of Lipid, glucose and unsaturated lipid of PMS rats were modulated by Zuogui-CSS, which were at the top of the VIP list (VIP > 1: significant).

Supplementary Figure 20.

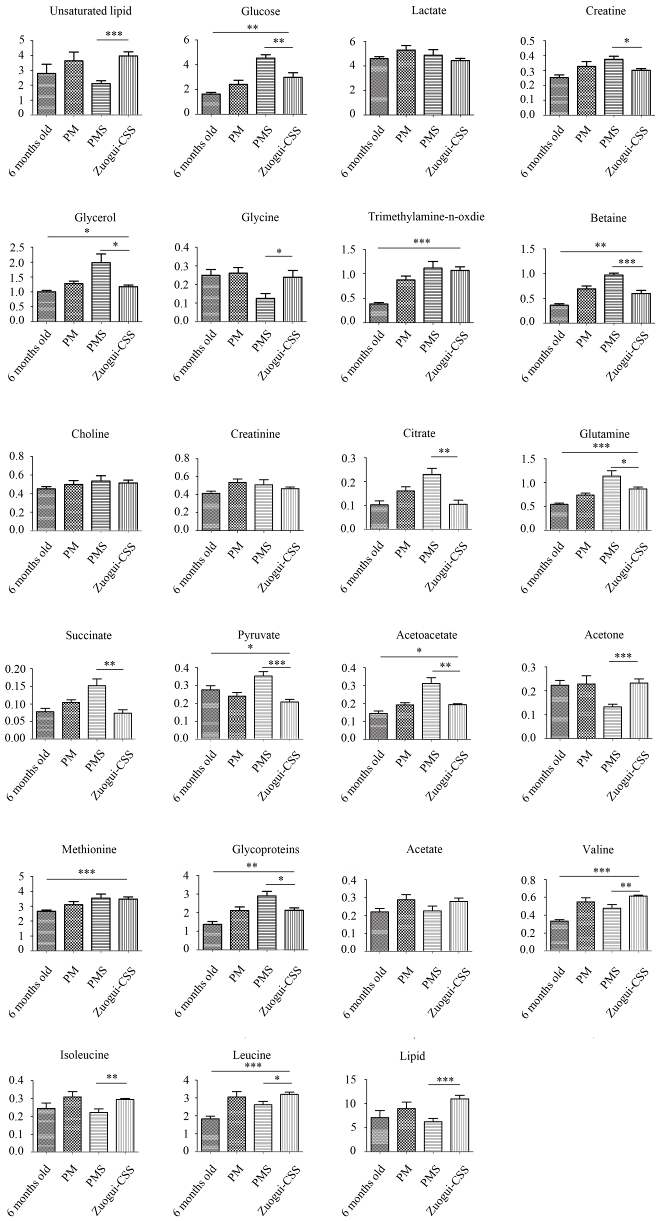

The combination treatment was effective. After Zuogui-CSS treatment, lipid metabolism was up-regulated and the metabolism of glucose was down-regulated. Zuogui-CSS treatment significantly lowered the levels of metabolites, such as glucose, creatine, glycerol, betaine, citrate, glutamine, succinate, pyruvate, acetoacetate, and glycoproteins, but the levels of unsaturated lipids, glycine, acetone, valine, isoleucine, leucine, and lipid were elevated. Zuogui-CSS treatment brought the levels of glycerol, glycine, citrate, succinate, and acetone to that of the 6-month-old rats. $p < 0.05$ (*); $p < 0.01$ (**); $p < 0.001$ (***).

Supplementary Table 6.

metabolites	Zuogui-CSS *vs* 6-month-old rats	Zuogui-CSS *vs* PMS rats
Unsaturated lipid		↑***
Glucose	↑**	↓**
Creatine		↓*
Glycerol	↑*	↓*
Glycine		↑*
Trimethylamine-n-oxide	↑***	
Betaine	↑**	↓***
Citrate		↓**
Glutamine	↑***	↓*
Succinate		↓**
Pyruvate	↓*	↓***
Acetoacetate	↑*	↓**
Acetone		↑***
Methionine	↑***	
Glycoproteins	↑**	↓*
Valine	↑***	↑**
Isoleucine		↑**
Leucine	↑***	↑*
Lipid		↑***

↑ indicates the increase and ↓ indicates the decrease of the metabolite concentrations in the plasma. $p<0.05$ (*); $p<0.01$ (**); $p<0.001$ (***).

Supplementary Figure 21.

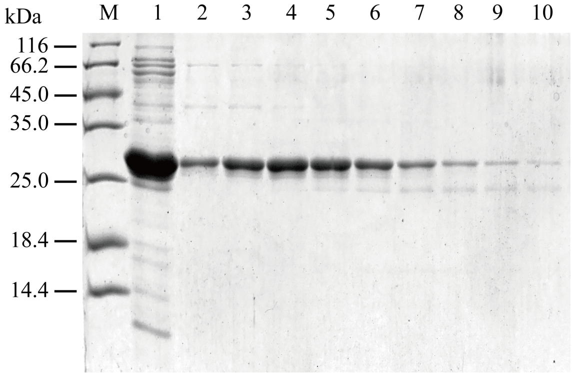

Purification of ERα-LBD. Purified proteins were analyzed by SDS-PAGE. The molecular weight markers were indicated, ERα-LBD had a molecular weight of approximately 30 kDa. Lane M, protein molecular weight markers; Lane 1, ERα-LBD eluted from an Ni-NTA affinity column; Lane 2 to Lane 10, Chromatographic purification of ERα-LBD in a Superdex 75 size-exclusion column. The proteins in Lanes 4 and 5 were used in the STD studies.
